# Proteomic networks associated with tumor-educated macrophage polarization and cytotoxicity potentiated by heat-killed tuberculosis

**DOI:** 10.1038/s41598-022-10463-x

**Published:** 2022-04-27

**Authors:** Denise U. Putri, Po-Hao Feng, Chiou-Feng Lin, Sofia M. Haryana, Marsetyawan H. N. E. Soesatyo, Kang-Yun Lee, Chia-Li Han

**Affiliations:** 1 International Ph.D. Program in Medicine, College of Medicine, Taipei Medical University, Taipei, 11031 Taiwan; 2 Doctorate Program of Medical and Health Science, Faculty of Medicine, Public Health and Nursing, Universitas Gadjah Mada, Yogyakarta, 55281 Indonesia; 3 Pulmonary Research Center, Wanfang Hospital, Taipei Medical University, Taipei, 11031 Taiwan; 4 Division of Pulmonary Medicine, Department of Internal Medicine, Shuang Ho Hospital, Taipei Medical University, New Taipei City, 23561 Taiwan; 5 Division of Pulmonary Medicine, Department of Internal Medicine, School of Medicine, College of Medicine, Taipei Medical University, 250 Wuxing St., Taipei, 11031 Taiwan; 6 Department of Microbiology and Immunology, School of Medicine, College of Medicine, Taipei Medical University, Taipei, 11031 Taiwan; 7 Department of Histology and Cell Biology, Faculty of Medicine, Public Health and Nursing, Universitas Gadjah Mada, Yogyakarta, 55281 Indonesia; 8 Graduate Institute of Clinical Medicine, College of Medicine, Taipei Medical University, Taipei, 11031 Taiwan; 9 Master Program in Clinical Genomics and Proteomics, College of Pharmacy, Taipei Medical University, 250 Wuxing St., Taipei, 11031 Taiwan

**Keywords:** Infection, Inflammation, Proteomics, Proteomics, Cell death and immune response, Bacterial infection, Tuberculosis, Lung cancer

## Abstract

Local administration of attenuated mycobacterium has been used as a cancer treatment adjuvant to re-boost patient immune responses with variable clinical outcomes. We aimed to clarify the impact of attenuated heat-killed tuberculosis (HKTB) on tumor-associated macrophages which play critical roles in shaping immunological regulation in the tumor microenvironment. Upon HKTB stimulation, both primary macrophages derived from the peripheral blood of healthy subjects and from lung cancer patients as well as THP1-derived classically activated macrophages (Ms) and tumor-educated macrophages (TEMs) were polarized into the proinflammatory phenotype, as characterized by increased expression cluster of differentiation 86. A quantitative proteomic analysis revealed that stimulated TEMs were unable to activate the toll-like receptor 2, signal transducer and activator of transcription 1, or nuclear factor-κB signaling. Instead, they showed distinct intercellular adhesion molecule 1 signaling, impaired cell adhesion, and mitochondrial dysfunction. These molecular mechanisms might contribute to lower cytotoxicity of HKTB-stimulated TEMs against A549 cells via the release of distinct inflammatory cytokines compared to HKTB-stimulated Ms. Our study provides an unbiased and systematic interpretation of cellular and molecular alterations of HKTB-reeducated macrophages which should help illuminate potential strategies of HKTB-stimulated macrophage-based combination therapy for cancer treatment.

## Introduction

Administration of attenuated bacteria is one of the oldest immunotherapeutics for cancer treatment^1^ with promising innate immune stimulation and cancer cell killing^2–6^. Following the approval of Bacille-Calmette-Guerin (BCG), an attenuated *Mycobacterium bovis*, by the US Food and Drug Administration as in situ bladder cancer immunotherapy^7^, researchers have been studying the therapeutic efficacies of BCG and other attenuated strains of mycobacteria in various cancers. BCG is also listed as an option for an intralesional injection for inoperable stage III in-situ melanomas with a high response rate^8^. BGC and other mycobacteria, including *M. brummae*, *M. phlei*, and *M. indicus pranii*, were studied for treatment of bladder cancer^9,10^, hepatoma^11^, and lung and cervical cancer cell lines^12^, while killed *M. vaccae* showed promise as adjuvant treatment for non-small-cell lung cancer^13,14^. Despite the remarkable risk reductions of disease progression, recurrence, and mortality shown in those preliminary studies, various responses to attenuated mycobacterial treatment were observed. Serious side effects and progressing infections were also detected^15,16^. From cellular and molecular points of view, these mycobacterium-based immunotherapies may operate through a direct cytotoxic effect against cancer cells and modulate surrounding immune systems by increasing tumoral antigenicity and activation of immune cells^3,17^. However, determining the precise ways in which they achieve their therapeutic effects requires further detailed investigations.

Macrophages are the first responders to pathological stimuli and the most abundant tumor-infiltrating antigen-presenting cells in the tumor microenvironment, critically shaping the composition of immune cells and regulating the kinetics of early immunological responses against tumors^18,19^. Due to their high plasticity, macrophages in the tumor microenvironment are dynamically manipulated by environmental stimuli to become the proinflammatory M1 phenotype for cancer cell killing or the anti-inflammatory M2 phenotype (termed tumor-associated macrophages) which facilitates tumor growth and metastases^20,21^. The coexistence and heterogeneous expressions of both phenotypes of macrophages in the tumor microenvironment have pivotal roles in modulating immunological reactions, making macrophages ideal targets for developing immunotherapeutics^22,23^. One of the approaches is to reprogram macrophage populations into more of the M1 phenotype through upregulation of histidine-rich glycoprotein^24^, activation of cluster of differentiation 40 (CD40)^25^, and stimulation of interferon (IFN)-α^26^. Nevertheless, there is insufficient evidence to explain how mycobacterial stimulation affects distinct phenotypes of macrophages within the tumor microenvironment. It is therefore important to explore the molecular effects of low-virulent mycobacteria to re-boost immune responses in heterogeneous macrophage populations which would provide insights into developing more-effective immunotherapies.

For this purpose, we aimed to evaluate cellular and molecular interactions between heat-killed tuberculosis (HKTB) and heterogeneous populations of pro- and anti-inflammatory macrophage phenotypes and study the cytotoxic potential and intracellular regulation of HKTB-stimulated macrophages. Specifically, we examined phenotype changes and secreted cytokine profiles as well as delineated regulatory mechanisms in HKTB-stimulated macrophages using quantitative proteomics analyses. Based on our findings, we proposed several dysregulated signaling pathways and molecules as targets in a search for combination treatments which may aid the potential use of HKTB as cancer immunotherapy.

## Results

### HKTB stimulation induced polarization of TEMs towards the proinflammatory phenotype

**Figure 1:**
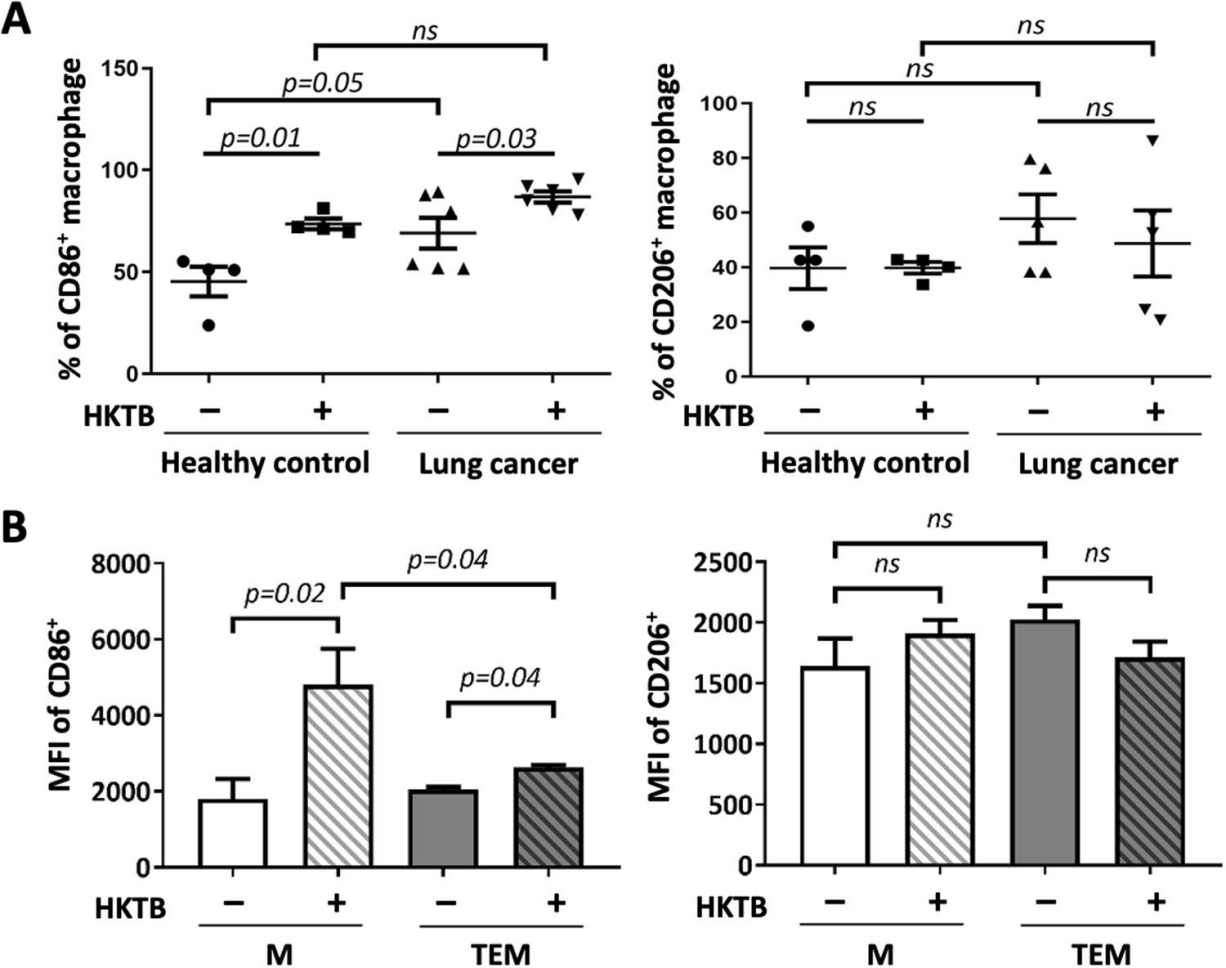
Flow cytometric analysis of macrophage phenotypes. Phenotypes of (**A**) primary peripheral blood macrophages from healthy subjects and lung cancer patients and (**B**) THP1-derived classically activated macrophage (M) and tumor-educated macrophage (TEM) models were determined before and after 3 days of heat-killed tuberculosis (HKTB) stimulation. Data with ≤ 50 cells within the gate were omitted. ns, not significant.

We collected blood samples from six lung cancer patients and four healthy subjects (Supplementary Table S1) and gated the peripheral blood macrophages as CD11b^+^HLA-DR^+^ cells (the gating strategy is shown in Supplementary Fig. S1A). After stimulation with 20 µg/mL HKTB for 72 h, the phenotypes of macrophages were evaluated by flow cytometry using the CD86 (proinflammatory M1) and CD206 (anti-inflammatory M2) markers (Supplementary Fig. S1A). As shown in Fig. 1A, stimulation with HKTB significantly induced expressions of CD86^+^ macrophages in both cancer and healthy groups without a significant change in the expression of CD206^+^ macrophages, indicating a promotion towards the proinflammatory phenotype.

A heterogeneous cancerous macrophage population was modeled in vitro from the co-culture of THP1-derived macrophages and A549 cancer cells, termed TEMs. THP1-derived classically activated macrophages (Ms) were also evaluated to serve as a reference for functional comparison. The gating strategy of flow cytometry for Ms and TEMs is presented in Supplementary Fig. S2A. The flow cytometric analysis verified that the M model constituted a higher fraction of CD86^+^ cells, while TEMs showed a higher CD206^+^ population (Supplementary Fig. S2B,C). After 72 h of HKTB stimulation, expressions of CD86^+^ macrophages had increased, while there was no significant change in expressions of CD206^+^ macrophages in either model (Fig. 1B). These results are consistent with observations of peripheral macrophages from lung cancer patients and healthy subjects. Macrophages from both lung cancer patients and THP1-derived TEMs were polarized into the pro-inflammatory phenotype after HKTB stimulation.

### Differential proteome profiles of TEMs upon HKTB stimulation

**Figure 2:**
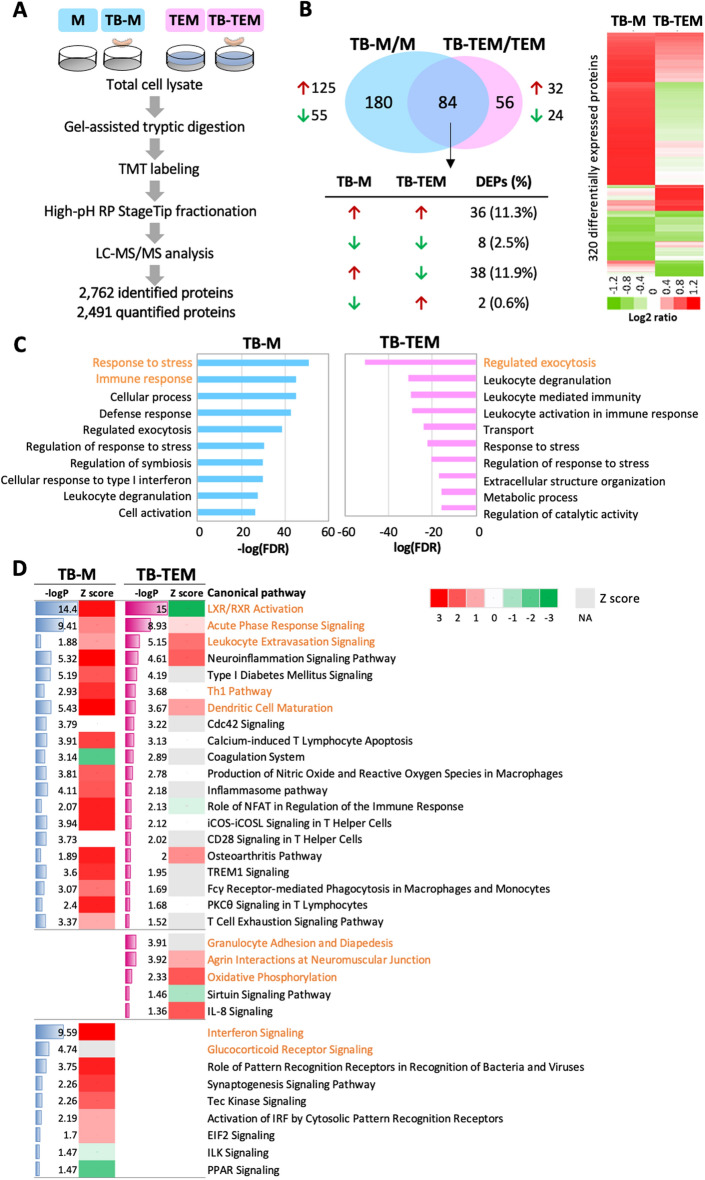
Differential proteomics profiles of heat-killed tuberculosis (HKTB)-stimulated classically activated macrophages (TB-Ms) and tumor-educated macrophages (TB-TEMs) with comparisons of Ms and TEMs. (**A**) Total cell lysates were collected for tandem mass tag (TMT)-based quantitative proteomics analysis to identify 2762 proteins, among which 2491 were quantified. (**B**) Overlap of differentially expressed proteins (DEPs) in the HKTB-stimulated M (TB-M) and TEM (TB-TEM) groups. (**C**) Enriched biological processes based on DEPs in the TB-M and TB-TEM groups using the Gene Ontology database. (**D**) Enriched pathways based on DEPs in the TB-M and TB-TEM groups using an Ingenuity Pathway Analysis (IPA). A z-score of > 0 indicates activation, while < 0 indicates inhibition of a cellular function or pathway. *NA* not available. Highlighted biological processes and pathways are marked in orange.

To explore the molecular mechanism of TEMs in response to HKTB stimulation, we analyzed the differential proteome expression profiles of HKTB-stimulated TEMs (shortened to TB-TEM) in reference to HKTB-stimulated Ms (shortened to TB-M). As shown in Fig. 2A, total cell lysates from the HKTB-treated and non-treated macrophage groups were collected for TMT-based quantitative proteomics analyses. In total, 2762 proteins were confidently identified (*p* < 0.05, FDR < 1%), among which 264 and 140 DEPs were respectively filtered into the HKTB-treated M and TEM groups. Among these, we observed 36 commonly upregulated and eight commonly downregulated proteins in both HKTB-stimulated groups. In addition, 40 proteins showed opposite regulatory directions; 38 proteins were upregulated in HKTB-treated Ms and downregulated in HKTB-treated TEMs, while two proteins showed reversed expressions. DEPs in the HKTB-stimulated M and TEM groups are listed in Supplementary Tables S2 and S3, respectively. Overall expression levels of DEPs are also given in Fig. 2B, showing distinct differential profiles in TEMs in response to HKTB.

We applied a Gene Ontology analysis of DEPs to determine the dysregulated biological processes in HKTB-stimulated macrophages. We first evaluated the basal level of the two untreated macrophage groups and observed the upregulations of myeloid cell response, phagocytosis, adhesion, and cytoplasm organization functions in TEMs compared to the Ms (Supplementary Table S4). Upon HKTB stimulation, as expected, several immune-related biological processes, including response to stress, immune response, defense response, leukocyte degranulation, cell activation, and cellular response to type I interferon, were dominantly enriched in HKTB-stimulated Ms (Fig. 2C, left panel). Despite the lower proinflammatory potential of TEMs in response to mycobacterial stimulation compared to classically activated macrophages, dysregulation of immune-related biological processes was also observed in TEMs after HKTB stimulation with additional dysregulation of the extracellular structure organization and metabolic processes (Fig. 2C, right panel). The IPA analysis further revealed common and unique dysregulated pathways (Fig. 2D). Several inflammatory-related pathways were enriched in both groups, including upregulation of acute phase response signaling, dendritic cell maturation, and leukocyte extravasation signaling pathways. However, LXR/RXR activation and NFAT in regulating immune responses were inhibited in TEMs after HKTB stimulation, but activated in HKTB-stimulated Ms. Exclusively, we identified cell type-specific pathways including interferon signaling and glucocorticoid receptor signaling in HKTB-stimulated Ms, as well as granulocyte adhesion and oxidative phosphorylation in HKTB-stimulated TEMs.

### Distinct molecular mechanisms underlying HKTB stimulation of Ms and TEMs

**Figure 3:**
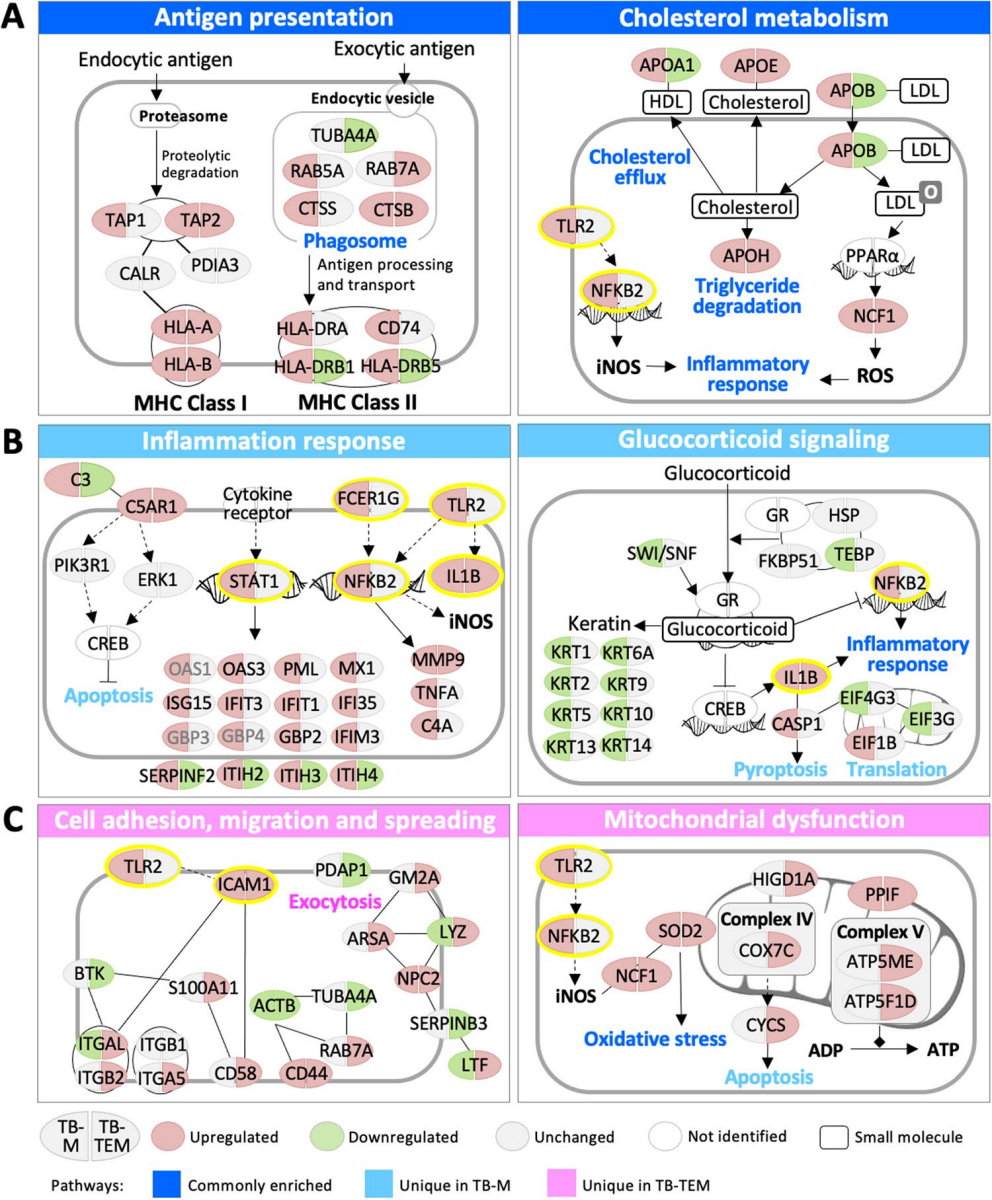
Unique and commonly dysregulated mechanisms in the heat-killed tuberculosis (HKTB)-stimulated classically activated macrophage (TB-M) and HKTB-stimulated tumor-educated macrophage (TB-TEM) models. (**A**) Commonly enriched mechanisms in both the TB-M and TB-TEM models. (**B**) Uniquely enriched mechanisms in TB-Ms. (**C**) Uniquely enriched mechanisms in TB-TEMs. Proteins with grey gene names were identified in a single-batch of experiments only.

Based on the dysregulated biological processes and pathways, we constructed molecular mechanisms in Ms and TEMs in response to HKTB stimulation. According to the dysregulation trend, we highlighted three categories of pathways in Fig. 3; those enriched in both HKTB-treated M and TEM groups are presented with a dark-blue ribbon (Fig. 3A), those uniquely enriched in HKTB-treated Ms are presented with a light-blue ribbon (Fig. 3B), and those uniquely enriched in HKTB-treated TEMs are shown with a pink ribbon (Fig. 3C). First, antigen processing and presentation pathways and cholesterol metabolism were commonly enriched in both groups but showed different regulatory mechanisms (Fig. 3A). We observed that MHC class I and II antigen processing and presentation pathways were upregulated in HKTB-stimulated Ms, as evidenced by the upregulation of endocytic antigen processing by transporters 1 and 2 of the ATP-binding cassette subfamily B (TAP1 and TAP2), exocytic antigen processing by Ras-related protein 5A (RAB5A), cathepsin S (CTSS), and CTSB, and antigen presentations by class I (HLA-A, HLA-B) and class II (HLA-DRA, HLA-DRB1, HLA-DRB5, and CD74) human leukocyte antigens. In contrast, only proteins involved in HLA class I antigen processing and presentation were consistently upregulated in HKTB-stimulated TEMs. HLA-DRB1 and HLA-DRB5 were downregulated, suggesting limited activation of downstream inflammatory reactions (Fig. 3A, left panel). Contrary to regulatory trends also observed in cholesterol metabolism, proteins involved in low-density lipoprotein (LDL) influx (apolipoprotein B, APOB), high-density lipoprotein (HDL) efflux (APOE and APOA1), and triglyceride degradation (APOH) were upregulated in HKTB-stimulated Ms, while APOA1 and APOB were downregulated in the HKTB-stimulated TEM model. The increase in intracellular lipids through cholesterol metabolism together with nuclear factor (NF)-κB signaling mediated increased oxidative stress within HKTB-stimulated Ms, as indicated by upregulation of neutrophil cytosolic factor 1 (NCF1), a downstream signal of peroxisome proliferator-activated receptor α (PPARα) (Fig. 3A, right panel).

Second, exclusive activation of immune-related signaling was observed in HKTB-stimulated Ms (Fig. 3B). Of note, complement 3 (C3) and C5a receptor 1 (C5AR1), which are involved in pathogen recognition, were upregulated. Toll-like receptor 2 (TLR2) and the Fc fragment of the immunoglobulin E (IgE) receptor (FCER1G) were upregulated, promoting NF-κB signaling and subsequent secretion of proinflammatory cytokines and chemokines including interleukin (IL)-1β, TNF-α-induced protein 8 (TNFAIP8), matrix metallopeptidase 9 (MMP9), and C4B. Upregulation of signal transducer and activator of transcription 1 (STAT1) by interferon signaling was uniquely observed in HKTB-stimulated Ms, which induced transcription of a series of proinflammatory mediators (OAS1, OAS3, PML, MX1, ISG15, IFIT3, IFIT1, IFI35, GBP3, GBP4, GBP2, and IFIM3) and proteins involved in acute phase response signaling (ITIH2, ITIH3, ITIH4, and SERPINF2).

In addition, we noted that glucocorticoid signaling, which was reported to inhibit the inflammatory response^27^, was downregulated in the HKTB-stimulated Ms (Fig. 3B, right panel). Downregulation of prostaglandin E synthase 3 (PTGES3, also named TEBP) and the SWI/SNF complex (represented by actin-β, ACTB) indicated inhibited glucocorticoid receptor (GR) signaling which lowered the transcription of a number of cytoskeletal keratins and directly or indirectly induced an inflammatory response through IL-1B and NF-κB signalling pathways. Furthermore, inhibition of glucocorticoid signaling reversibly activated CREB transcription and then promoted caspase-1 (CASP1)-mediated apoptosis signaling. Proteins involved in the translation process, including eukaryotic translation initiation factor 1B (EIF1B), EIF3G, and EIF4G3, were also enriched in HKTB-stimulated Ms, although the net effect was unclear.

Third, we observed that activated cell adhesion, migration, and spread as well as exocytosis and mitochondrial dysfunction were exclusively enriched in HKTB-stimulated TEMs (Fig. 3C). Upon sensing pathogens, migration and adhesion of macrophages to sites of infection are indispensable for immune responses^28^. CD44, an adhesion molecule that serves as an important binding site of mycobacteria to macrophages, was upregulated in both the M and TEM models which would promote macrophage recruitment^29^. Cell adhesion-related proteins, including integrin complexes (ITGB2, ITGA5, and ITGAL), intracellular adhesion molecule 1 (ICAM1), and CD58, were mainly upregulated in HKTB-stimulated TEMs (Fig. 3C, left panel). Moreover, proteins that participate in exocytosis which mediates antigen processing were enriched with upregulation of arylsulfatase A (ARSA), GM2 ganglioside activator (GM2A), lysozyme (LYZ), and NPC intracellular cholesterol transporter 2 (NPC2). Proteins involved in mitochondrial dysfunction were also uniquely enriched in HKTB-stimulated TEMs (Fig. 3C, right panel), especially those in the oxidative phosphorylation complexes IV and V which produce ATP and induce apoptosis. NCF1- and superoxide dismutase 2 (SOD2)-mediated oxidative stress increased in both HKTB-stimulated M and TEM models.

### Functional validation

**Figure 4:**
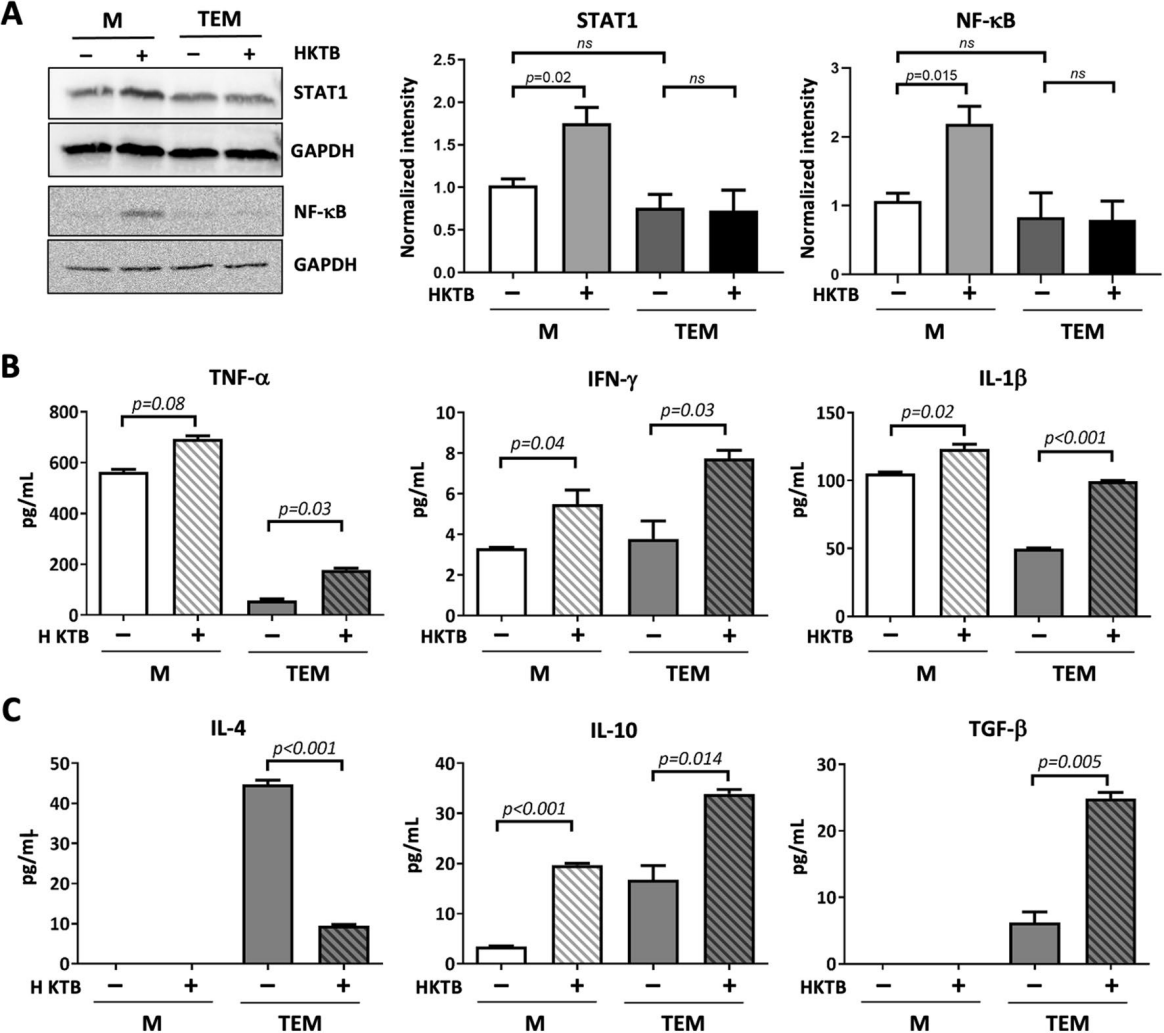
Functional validation. (**A**) Representative Western blot images and statistical results for expression levels of signal transduction and activator of transcription 1 (STAT1) and nuclear factor (NF)-κB in heat-killed tuberculosis (HKTB)-stimulated classically activated macrophages (Ms) and tumor-educated macrophages (TEMs) before and after HKTB stimulation. GAPDH was used as an internal control. Measurement of levels of (**B**) proinflammatory cytokines and (**C**) anti-inflammatory cytokines released by Ms and TEMs before and after HKTB stimulation. Statistical analyses were performed by Student's *t*-test. ns, not significant.

STAT1 and NF-κB, the main molecules modulating inflammatory reactions through transcriptional regulation of the production of acute-phase response proteins as well as pro- and anti-inflammatory cytokines in HKTB-stimulated macrophages (Fig. 3B), were selected for Western blot validation. As shown in Fig. 4A and Supplementary Fig. S3, higher STAT1 and NF-κB expressions in the HKTB-stimulated M model were observed, while there were no significant changes in expressions of either protein in the HKTB-stimulated TEM model. In addition, the M model secreted proinflammatory TNF-α, IFN-γ, and IL-1β (Fig. 4B), and a low level of anti-inflammatory IL-10 in the absence of HKTB stimulation (Fig. 4C). After 72 h of HKTB stimulation, levels of proinflammatory cytokines in Ms significantly increased along with IL-10 secretion. On the contrary, untreated TEMs constitutively released lower levels of TNF-α and IL-1β and higher levels of the anti-inflammatory IL-4, IL-10, and TGF-β compared to Ms. HKTB stimulation induced significant secretion of proinflammatory IFN-γ, IL-1β, and TNF-α as well as the anti-inflammatory IL-10 and TGF-β while IL-4 was suppressed. In summary, HKTB potentiated both the proinflammatory and anti-inflammatory functions of TEMs.

### HKTB stimulation potentiated cytotoxicity of Ms and TEMs towards lung cancer cells

**Figure 5:**
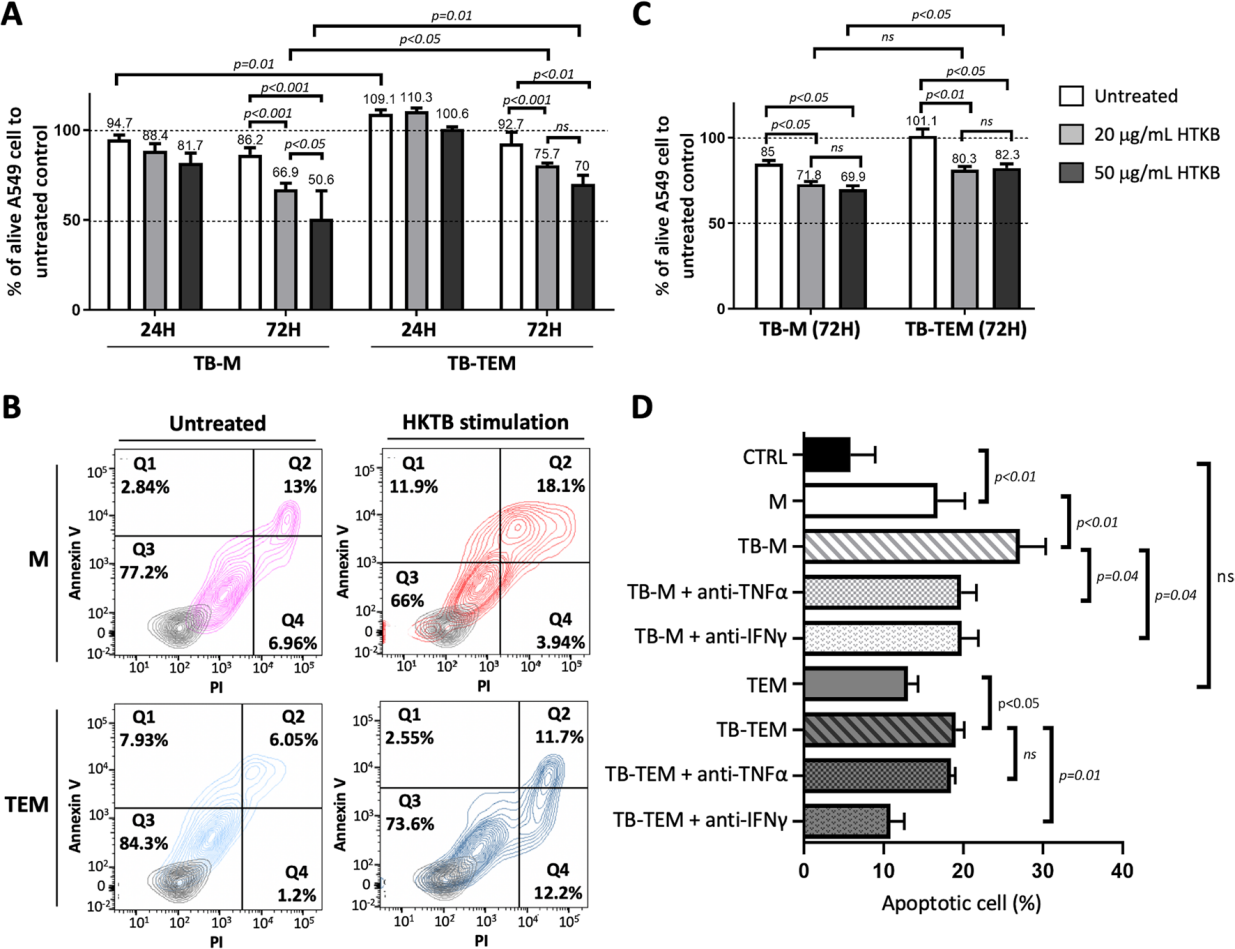
Cytotoxicity analysis of heat-killed tuberculosis (HKTB)-stimulated classically activated macrophages (Ms) and tumor-educated macrophages (TEMs) against A549 cells. (**A**) An MTT assay was used to determine A549 cell viability upon coculture with Ms and TEMs as well as HKTB-stimulated Ms (TB-M) and TEMs (TB-TEM) stimulated with 20 or 50 mg/mL HKTB for 24 or 72 h, respectively. (**B**) Flow cytometric analysis of A549 cells showed apoptotic and necrotic cells within the cell population. (**C**) The MTT assay determined A549 cell viability upon culturing with medium from Ms and TEMs and from TB-M and TB-TEM stimulated with 20 or 50 mg/mL HKTB for 72 h. (**D**) The flow cytometric assay determined A549 cell viability with or without the inhibition of tumor necrosis factor (TNF)-α and INF-γ. ns, not significant.

In order to specifically address the potential of HKTB in promoting macrophage cytotoxicity towards cancer cells, we applied 20 and 50 µg/mL of HKTB for co-culture with Ms and TEMs and observed the cytotoxicity of HKTB-stimulated Ms and TEMs towards A549 cells by assessing cell viability and apoptosis/necrosis. The dosages were selected based on the results from direct treatment of HKTB on A549 or THP1-derived macrophages which did not show significant cell death, even up to 80 µg/mL (Supplementary Fig. S4). As shown in Fig. 5A, significant death of A549 cells was observed after 72 h of co-culturing with both HKTB-stimulated Ms and TEMs, but no significant cell death was observed upon short-term (24 h) co-culturing. We further differentiated the cell death mechanism and observed 15.8% and 30% apoptosis and 7% and 3.9% necrosis in A549 cells upon 72 h of coculture with the HKTB-stimulated M and TEM models, respectively (Fig. 5B). In summary, 72 h of coculturing with HKTB-stimulated M and A549 cells significantly reduced cancer cell viability with more cells undergoing apoptosis in a dose-dependent manner. In HKTB-stimulated TEMs, we observed a significant cancer cell-killing effect after 3 days of co-culturing with no difference between 20 and 50 µg/mL of HKTB.

The co-culture system prevents direct interactions between cells, suggesting the role of secreted cytokines in mediating the cytotoxicity of HKTB-stimulated macrophages towards cancer cells. As shown in Fig. 5C, A549 cells cultured with pre-stimulated medium from both HKTB-stimulated Ms and TEMs exhibited significant increases in cell death, although the net cytotoxic effects were less robust compared to observations in the coculture system (Fig. 5A). There were no significant differences in A549 cell viability between treatments by pre-stimulated medium from the M and TEM models using stimulation with either 20 or 50 µg/mL HKTB. Additionally, the cytotoxic activity did not differ between 20 µg/mL HKTB stimulation in the two models. These results indicated that the cytotoxicity toward A549 cells might be partly from released proinflammatory cytokines from HKTB-stimulated Ms and TEMs. Inhibition of TNF-α and IFN-γ further revealed a significant reduction in cytotoxic activity in the HKTB-stimulated M model to an extent similar to the untreated model. Meanwhile, only inhibition of IFN-γ in the HKTB-stimulated M model showed loss of cytotoxicity (Fig. 5D).

## Discussion

**Figure 6:**
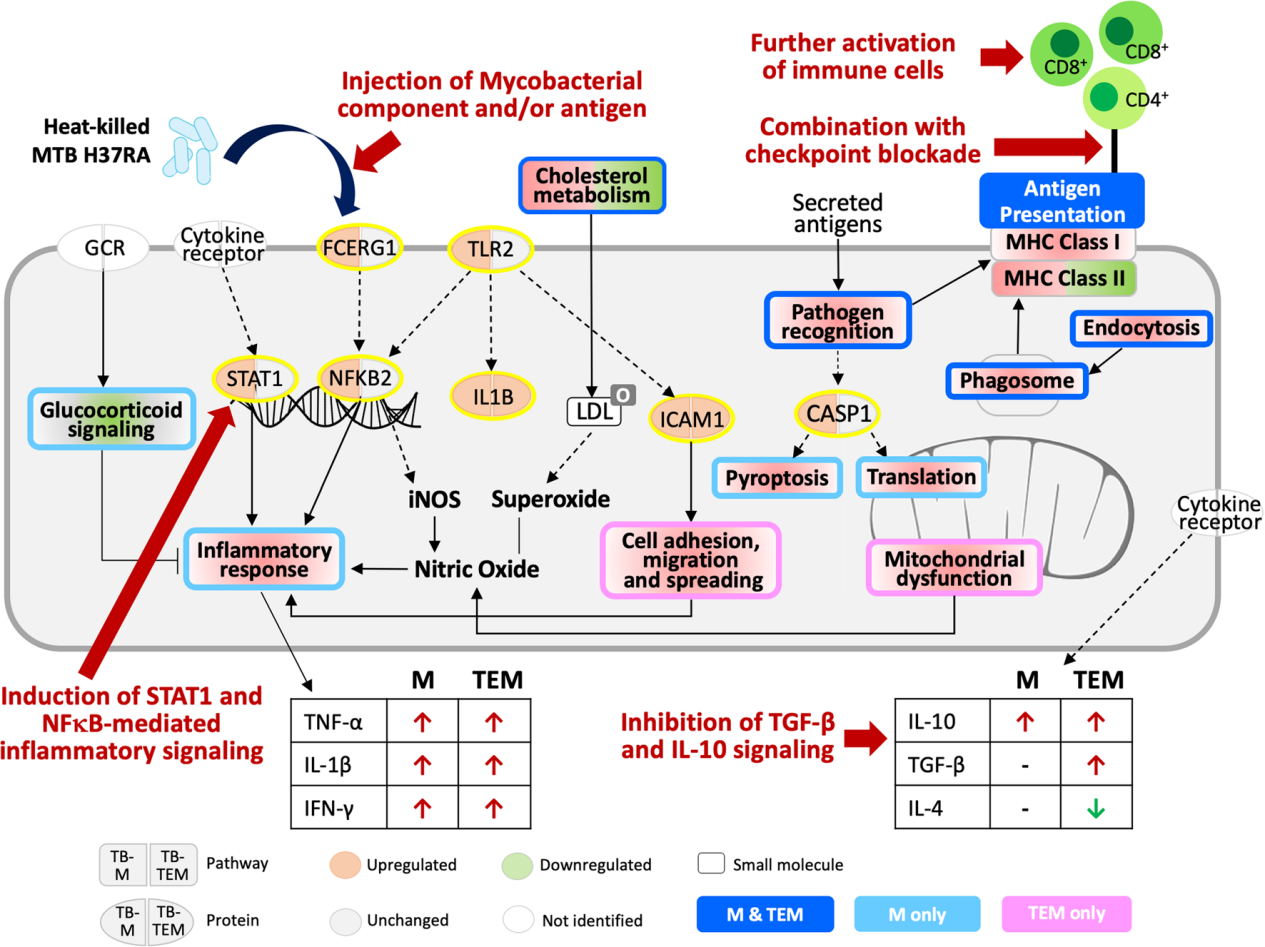
Summarized molecular mechanisms in heat-killed tuberculosis (HKTB)-stimulated classically activated macrophage (M) and tumor-educated macrophage (TEM) models. Based on the mechanism, we proposed alternative approaches to enhance the cancer immunotherapy using attenuated mycobacteria, as indicated by red arrows.

In cancer immunotherapy, an apparent switch from the pro-tumoral M2 to the antitumoral M1 phenotype is crucial to re-boost the immune response and enhance the cytotoxicity of TEMs towards cancer cells^30,31^. This transition can be initiated by reprogramming and/or replacement of TEMs via recruiting and differentiating monocytes into M1 macrophages under the influence of a newly established microenvironment^32^. However, the detailed regulatory mechanism remains unclear. In the present study, we observed that HKTB promoted the proinflammatory phenotype in TEMs in the peripheral blood of lung cancer patients and the THP1-derived macrophage models. However, several key inflammation-associated proteins, including TLR2, STAT1, NF-κB, and FCER1G (marked in the yellow circle in Figs. 3 and 6), were not regulated upon HKTB stimulation in TEMs, but IL-1B and ICAM1 were regulated. Downregulation of glucocorticoid signaling was also not observed in HKTB-stimulated TEMs. With mycobacterial infection, activation of the TLR2-mediated NF-κB and STAT1 pathways was reported to promote proinflammatory macrophages by M1 polarization and production of proinflammatory cytokines^33–35^, attenuation of M2 activity^36^, and induction of macrophage apoptosis^37,38^. Downregulation of glucocorticoid signaling may lead to inhibition of protumoral M2 macrophage polarization and promotion of proinflammatory cytokines^27,39^, which is a novel observation in mycobacterial stimulation. The failure to activate or inhibit these signaling pathways may have contributed to the partial polarization of TEMs to the proinflammatory phenotype and lowered the cytotoxicity of HKTB-stimulated TEMs.

Together with the upregulated proinflammatory IL-1B and ICAM1, we observed exclusively regulated proteins involved in cell adhesion and mitochondria dysfunction which may facilitate chemotaxis and inflammation in HKTB-stimulated TEMs. ICAM1 was reported to inhibit M2 polarization^40^ and to mediate mycobacterial entry into macrophages together with integrins^41,42^. In addition, mitochondrial dysfunction is known to be induced by TGF-β^43^ and leads to defective macrophage phagocytosis^44^. This could induce activation of caspase proteins and NF-κB by oxidative stress or apoptotic pathways, and consequently release inflammatory mediators such as TNF-α and nitric oxide^45^. Van den Bossche et al*.* reported that M1-associated inhibition of mitochondrial oxidative phosphorylation is a key factor preventing M1 to M2 repolarization. They noted that the M2 phenotype exhibited higher plasticity in adopting an M1-like inflammatory state, while in contrast, M1 macrophages were steadier^46^. Hyporesponsiveness of TLR-NF-κB to HKTB stimulation may also have been caused in part by the overexpression of integrin β2 (ITGB2) in TEMs^47^.

In the present study, we detected increased levels of IL-10 and TGF-β in response to HKTB stimulation of TEMs, although it remains unclear whether both anti-inflammatory cytokines were secreted by the macrophages or cancer cells due to our co-culture system. IL-10 and TGF-β may attenuate secretion of proinflammatory cytokines by activated macrophages during *Mycobacterium tuberculosis* infection or upon lipopolysaccharide stimulation^48,49^, which are detrimental to the host. On the other hand, these cytokines are important in limiting tissue damage^50^. Furthermore, IL-10 was documented to suppress MHC class II expression^51^, as also observed in HKTB-stimulated TEMs in our study. To the present, no study has reported their roles in response to avirulent or heat-killed mycobacteria. Taken together, we speculated that the lower cytotoxic activity against cancer cells by HKTB-treated TEMs compared to HKTB-treated Ms might be a result of concomitant regulation between the high IL-10 and TGF-β levels and limited activation of inflammatory signaling.

Our study adopted crude preparations of heat-killed mycobacteria for co-culturing with THP1-derived macrophage models, which may have caused unexpected events due to complexity of the bacteria. Due to the limited number of peripheral macrophages that could be isolated for further assays, most of the analyses in the present study adapted in vitro THP1-derived macrophage models, which cannot accurately represent the overall interplay among HKTB, macrophages, and cancer cells in the tumor microenvironment. The cytotoxic effect of HKTB-stimulated macrophages should also be tested on other types of lung cancer cells. The phagocytic capacity of macrophages and direct contact studies between cancer cells and macrophages may be important and require further studies. In addition, macrophages in patients’ PBMC might not have the same phenotype as those in the tumor microenvironment. Further validation of the macrophage phenotypes in the tumor microenvironment and expression levels of inflammatory-associated mechanisms should be addressed in a greater number of patients to draw general conclusions about HKTB-stimulated regulation.

In summary, we reported the molecular regulation underlying HKTB-reeducated macrophages and propose several alternative approaches to improve the cytotoxicity of TEMs using attenuated mycobacteria in Fig. 6. As shown in Fig. 6, further study should aim to isolate certain mycobacterial components or agonists that mimic mycobacterial signals^52^ as potential immunotherapeutic strategies. We also suggest a new perspective for combination therapy to promote potential immunotherapeutic benefits of mycobacterial components/antigens. Of note, agents that limit the anti-inflammatory cytokines IL-10 and TGF-β, or activate TLR2, FECR1G, STAT1, and NF-κB, may be used to boost inflammatory responses of TEMs. Additionally, further activation of immune cell cascades warrants more profound studies as proper antigen presentation and lymphocyte activation are pivotal factors in the success of immunotherapies^53^. Combination therapy that targets both innate and adaptive immunity by improving the functions of antigen-presenting cells, notably macrophages, as well as cellular immunity cascade activation may serve as a powerful immunotherapeutic strategy as they induce more-comprehensive potentiation of immune cells^23,54^.

## Methods

### Isolation and culture of peripheral blood mononuclear cells (PBMCs)

PBMCs were collected from six lung cancer patients and four healthy subjects. This study was approved by the Taipei Medical University Joint Institutional Review Board, and informed consent was obtained from all subjects. All experiments were performed in accordance with relevant guidelines and regulations. Among the lung cancer patients (Supplementary Table S1), five had adenocarcinomas (83.3%) and one patient was diagnosed with squamous cell carcinoma (16.7%).

Venous blood was drawn and buffy coats were obtained through separation using SepMate tubes (STEMCELL Technologies, Vancouver, Canada) according to the manufacturer’s instructions. In brief, heparinized blood was diluted with phosphate-buffered saline (PBS), layered on top of Lymphoprep, and centrifuged at 1200×*g* for 10 min. PBMCs were collected, washed twice with PBS, and counted using a hemocytometer with trypan blue (Lonza, Basel, Switzerland) to determine cell viability. PBMCs were cultured in the same conditions as THP1 and A549 cells in the following section. After 24 and 72 h of culture, cells were washed, and adherent cells were collected to observe macrophage polarization using a flow cytometric assay.

### THP1-derived classically activated macrophages (Ms) and tumor-educated macrophages (TEMs)

An in vitro study was developed utilizing the THP1 human monocytic cell line (ATCC® TIB-202™, ATCC, Manassas, VA, USA) and the A549 lung adenocarcinoma cell line (ATCC® CCL-185™) for macrophage polarization, cytokine, and cell viability assays. Approximately 2 × 10^6^ THP1 and A549 cells were seeded in 10-cm dishes in filter-sterilized RPMI-1640 medium, 10% heat-inactivated fetal bovine serum (Gibco, Waltham, MA, USA), and 100 U/mL penicillin–streptomycin (Gibco). Cells were grown in a humidified incubator at 37 °C under 5% CO_2_. Routine subculturing was conducted every 3 days. Cells between passages two and eight were used for experiments. For differentiation, 2 × 10^6^ THP1 cells were seeded in 10-cm dishes and treated with 80 nM phorbol 12-myristate 13-acetate (PMA, Sigma Aldrich, St. Louis, MO, USA) for 24 h. For Ms, culture media were replaced with RPMI-1640 media and incubated for 24 h. To prepare TEMs, THP1-derived macrophages were further co-cultured with A549 cells in 0.4-µm polycarbonate permeable trans-well dishes (Corning, Corning, NY, USA) for 24 h.

### Heat-killed tuberculosis (HKTB) stimulation

Heat-killed *Mycobacterium tuberculosis* (HKTB) avirulent H37Ra strain (InvivoGen, San Diego, CA, USA) was prepared according to manufacturer’s instructions and used to stimulate Ms and TEMs, either in transwell chambers or tissue culture dishes, for 24 or 72 h in a humidified incubator at 37 °C under 5% CO_2_. Prior to harvesting, cells were washed with PBS and adherent cells were collected.

### Flow cytometry

Human peripheral blood macrophages and THP1-derived macrophages were submitted to flow cytometric analysis according to the manufacturer’s recommendations. After being washed with PBS, cells were stained with a LIVE/DEAD™ fixable blue dead cell stain kit (Invitrogen, Paisley, UK), followed by blocking of Fc receptors using Human TruStain FcX (BioLegend, San Diego, CA, USA) and cell surface labeling by a specific antibody or isotype control. Antibodies against CD11b, CD86, CD206, CD14, and human leukocyte antigen (HLA)-DR were used for the human peripheral blood macrophages, while antibodies against CD86, CD206, CD14, HLA-DR and CD68 were used for THP1-derived. All these antibodies were purchased from BioLegend (San Diego, CA, USA). Samples were washed once and analyzed in a BD LSRFortessa cytometer (BD Bioscience, San Jose, CA, USA) and FlowJo™ (v10, LLC, Ashland, OR, USA). Results are presented as the percentage of positive cells or as the ratio of the mean of fluorescence intensity (MFI) of the antibody of interest to the isotype control. Three technical replicate analyses were performed for human peripheral blood macrophages, while three biological and three technical replicate analyses were performed for THP1-derived macrophages.

### Tandem mass tag (TMT)-based quantitative proteomics analysis

THP1-derived M and TEM cells and the HKTB-stimulated M (TB-M) and TEM (TB-TEM) cells were first washed three times with PBS and scraped into lysis buffer containing 6 M urea, 5 mM ethylenediaminetetraacetic acid (G-Biosciences; St. Louis, MO, USA), 2% sodium dodecylsulfate, and 0.1 M triethylammonium bicarbonate buffer (TEABC, Sigma Aldrich, St. Louis, MO, USA). A protease inhibitor cocktail (Calbiochem, San Diego, CA, USA) was added in a volume ratio of 100:1 (sample: protease inhibitor, v/v, Calbiochem), followed by sonication at 4 °C for 15 min (Bioruptor, Diagenode, Belgium) and 30 min of centrifugation at 13,000×*g*. The supernatant was collected and quantified using a Pierce Bicinchoninic Acid (BCA) assay (Thermo Fisher Scientific, Rockford, IL, USA) to determine the protein concentration. Fifty micrograms of proteins were subjected to our previously reported gel-assisted digestion^55^. The resulting peptide was concentrated in a SpeedVac (Thermo Fisher Scientific) and resuspended in 100 mM TEABC for the BCA assay. Five micrograms of peptides from each sample were taken for labeling with TMT isobaric reagents (Thermo Fisher Scientific) following the manufacturer’s instructions. Untreated and HKTB-treated Ms were labeled with TMT127 and TMT128, respectively, while untreated and HKTB-treated TEMs were labeled with TMT129 and TMT130, respectively.

TMT-labeled peptides were pooled and fractionated using high-pH reverse-phase StageTip to collect six fractions following a previous study^56^. Each fraction was desalted by C18 ZipTip (Millipore, Cambridge, Ontario, Canada) and resuspended in mobile phase buffer A (0.1% formic acid in H_2_O) for subsequent LC–MS/MS analysis on a nanoAcquity system (Waters, Milford, MA, USA) connected to an LTQ-Orbitrap Velos (Thermo Fisher Scientific) equipped with a Nanospray Flex interface. Briefly, peptide mixtures were loaded onto a 75-μm i.d. × 25-cm C18 BEH column (Waters) packed with 1.7-μm particles with a pore size of 130 Å and separated using a segmented gradient from 1 to 45% of mobile phase buffer B (0.1% formic acid in acetonitrile) for 103 min at a constant flow rate of 0.3 µL/min and a column temperature of 35 °C. Peptides were detected in the data-dependent acquisition mode. Full-scan MS spectra were acquired in the orbitrap (*m/z* 350 to 1600, resolution 60,000 at *m/z* 400). The top 10 most intense ions with at least two positive charge states were sequentially isolated (with an isolation window of 2 Da and automatic gain control of 5E5) and fragmented by high-energy collision-induced dissociation in a multipole collision cell with a normalized collision energy of 45%. Fragmented ions were detected in the linear ion trap to acquire the MS/MS spectra.

For the proteomic analysis, three independent biological replicates and two technical replicates were analyzed, and proteins were identified by searching MS raw files against the SwissProt human protein sequence database (release 2019_02, 20,335 entries) using Mascot implemented in Proteome Discoverer (vers. 2.2.0.388, Thermo Fisher Scientific). Only tryptic peptides with up to two missed cleavages were allowed. The mass tolerances for precursor and fragment ions were set to 10 ppm and 0.1 Da, respectively. Methylthio (Cys) was set as a fixed modification, whereas oxidation (Met), acetylation (protein N-terminal), deamidation (Asn, Gln), and TMT tags (N-terminal, Lys) were set as variable modifications. Peptide-spectrum matches were validated using percolator (q-value of 1%). Peptide-spectrum matches and proteins with a < 1% false discovery rate were considered positive identifications.

For proteome quantitation, only master proteins with at least one unique peptide were quantified, and protein ratios were normalized by total peptide abundances. Based on our previous study^57^, we considered proteins with log2 ratios (TB-M/M or TB-TEM/TEM) of > 0.3785 or < -0.3785 (indicating a 1.3-fold difference in abundance) as differentially expressed proteins (DEPs).

### Functional enrichment analysis of DEPs

DEPs identified in at least two biological replicates were submitted to Gene Ontology (release 2019-06-01)^58,59^ for enrichment of dysregulated biological processes and to Ingenuity Pathway Analysis (IPA)^60^ for pathway enrichment analysis. Z-scores were obtained from the IPA for enriched canonical pathways and biofunctional annotations. Only annotations with *p* < 0.05 were considered significant hits. A z-score of > 0 indicates activation, while one of < 0 indicates inhibition of a cellular function or pathway. Unsupervised hierarchical clustering of proteome expression profiles was performed with Gene Cluster 3.0 vers. 1.52^61^, and visualized with Java Treeview vers. 1.1.6r4^62^.

### Western blot analysis

Twenty-μg of protein was run in gel electrophoresis by using 4–20% Mini-PROTEAN® TGX™ Gel (Bio-Rad, CA, USA), and transferred onto a nitrocellulose membrane. The membranes were blocked for one hour at room temperature and incubated overnight at 4 °C with primary antibodies against Nfkb p65, Stat1, and Gapdh (Abcam, MA, USA) at a:1000 concentration. Following three times washing step using phosphate-buffered saline with Tween-20, the membranes were incubated with the antirabbit or antimouse IgG secondary antibody in a concentration of 1:10,000 (Bioss Antibodies, MA, USA) for 1 h at room temperature. Clarity™ Western ECL Substrate was used to detect protein bands. Gapdh was used as the loading control, and three replicates of experiments were performed to obtain statistical significance. The detected protein bands were quantified using AzureSpot (Azure Biosystems, CA, USA).

### Quantification of cytokine levels by an enzyme-linked immunosorbent assay (ELISA)

Cytokine levels in culture media were quantified using an ELISA Ready-SET-Go kit (Ebioscience, San Diego, CA, USA) according to the manufacturer’s instruction. Proinflammatory tumor necrosis factor (TNF)-α, IFN-γ, and interleukin (IL)-1β cytokines and anti-inflammatory IL-4, IL-10, and transforming growth factor (TGF)-β cytokines were assayed. Samples were run in triplicate and quantified using a standard curve.

### Inhibition of IFN-γ and TNF-α

Neutralization antibodies against IFN-γ (20 µg/mL; Ebioscience, San Diego, CA, USA) and TNF-α (10 µg/mL; R&D Systems, Minneapolis, MN, USA) were added to THP1-derived macrophages for 1 h, followed by 72 h of HKTB stimulation. Pre-stimulated macrophages were then cocultured with A549 cells to determine their cytotoxic capability.

### Cell viability assay of A549 cells

A549 cells (10^5^) were seeded in the bottom chamber of six-well plates and cultured overnight. HKTB-stimulated or non-stimulated macrophages were pre-prepared in 0.4-µm polycarbonate permeable trans-well dishes (Corning) and respectively cocultured with A549 cells for 24 and 72 h. A549 cell viability was determined using an MTT Cell Growth Assay Kit (Merck Millipore, Darmstadt, Germany) according to the manufacturer’s instructions. To determine the cell death profile, A549 cells were also subjected to staining with a FITC Annexin V Apoptosis Detection Kit with propidium iodide (BioLegend, San Diego, CA, USA) according to the manufacturer’s instructions. At least three replicates of samples were submitted for each assay.

### Statistical analysis

Data obtained from macrophage polarization experiments and cytokine assays were analyzed by Student’s *t*-test. Cell viability results were analyzed by a two-way analysis of variance (ANOVA) followed by a post-hoc multiple-comparison test. Data graphs are presented in mean values with standard deviations and generated with GraphPad Prism (La Jolla, CA, USA). For all statistical analyses, results with *p* < 0.05 were considered significant.

## Supplementary Information

Supplementary Information.

## Data Availability

The datasets generated during and/or analyzed during the current study are available from the corresponding author on reasonable request.
