# Supplementary material for: Proteomic networks associated with tumor-educated macrophage polarization and cytotoxicity potentiated by heat-killed tuberculosis
Source: Sci Rep. 2022 Apr 27;12:6881. doi: 10.1038/s41598-022-10463-x (PMC9046162; doi:10.1038/s41598-022-10463-x)
Supplement: Supplementary file 1 — Supplementary Information. [file 41598_2022_10463_MOESM1_ESM.pdf]

Supplementary Materials

Proteomic Networks Associated with Tumor-Educated Macrophage Polarization and Cytotoxicity Potentiated by Heat-Killed Tuberculosis

Denise U. Putri[1.2,3], Po-Hao Feng[4,5], Chiou-Feng Lin[6], Sofia M. Haryana[7], Marsetyawan HNE. Soesatyo[7], Kang-Yun Lee[4,5,8*], Chia-Li Han[9*]

[1] International Ph.D. Program in Medicine, College of Medicine, Taipei Medical University, Taipei, Taiwan

[2] Doctorate Program of Medical and Health Science, Faculty of Medicine, Public Health and Nursing, Universitas Gadjah Mada, Yogyakarta, Indonesia

[3] Pulmonary Research Center, Division of Pulmonary Medicine, Department of Internal Medicine, Wanfang Hospital, Taipei Medical University, Taipei, Taiwan

[4] Division of Pulmonary Medicine, Department of Internal Medicine, Shuang Ho Hospital, Taipei Medical University, New Taipei City, Taiwan

[5] Division of Pulmonary Medicine, Department of Internal Medicine, School of Medicine, College of Medicine, Taipei Medical University, Taipei, Taiwan

[6] Department of Microbiology and Immunology, School of Medicine, College of Medicine, Taipei Medical University, Taiwan

[7] Department of Histology and Cell Biology, Faculty of Medicine, Public Health and Nursing, Universitas Gadjah Mada, Yogyakarta, Indonesia

[8] Graduate Institute of Clinical Medicine, College of Medicine, Taipei Medical University, Taipei, Taiwan

[9] Master Program in Clinical Genomics and Proteomics, College of Pharmacy, Taipei Medical University, Taipei, Taiwan

* Corresponding authors

List of Supplementary Materials

Supplementary Fig. S1. The gating strategy and phenotype analysis of peripheral blood macrophages from healthy subjects and lung cancer patients by flow cytometry analysis.

Supplementary Fig. S2. The gating strategy and phenotype analyses of THP1-derived macrophages upon 3-days stimulation with HKTB.

Supplementary Fig. S3. The images of western blot experiments.

Supplementary Fig. S4. Viability analysis of A549 cells and THP1-derived macrophages.

Supplementary Table S1. Clinical characteristics of recruited subjects.

Supplementary Table S2. Differentially expressed proteins in TB-M group.

Supplementary Table S3. Differentially expressed proteins in TB-TEM group.

Supplementary Table S4. Enriched biofunctions in untreated TEM in comparison with untreated M.

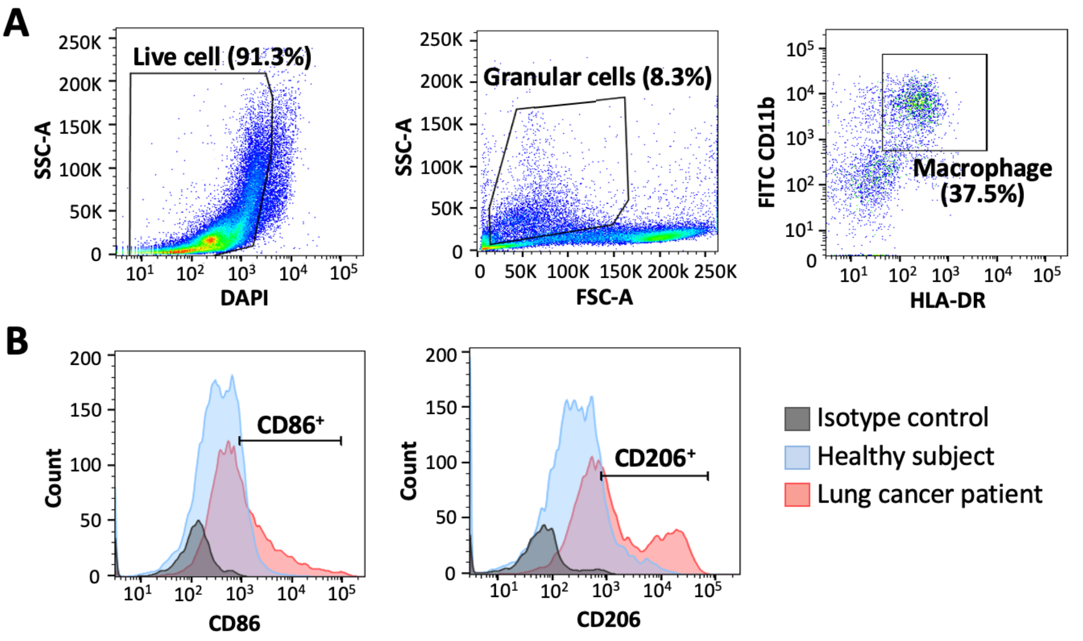

Supplementary Fig. S1. The gating strategy and phenotype analysis of peripheral-blood macrophages from healthy subjects and lung cancer patients by flow cytometry analysis. (A) Macrophages were gated as live, granular cells with high CD11b^{+}/HLA-DR^{+} expressions. **(B)** Distribution of CD86^{+} and CD206^{+} macrophages in healthy subject (blue) and lung cancer patient (red) in comparison to isotype control.

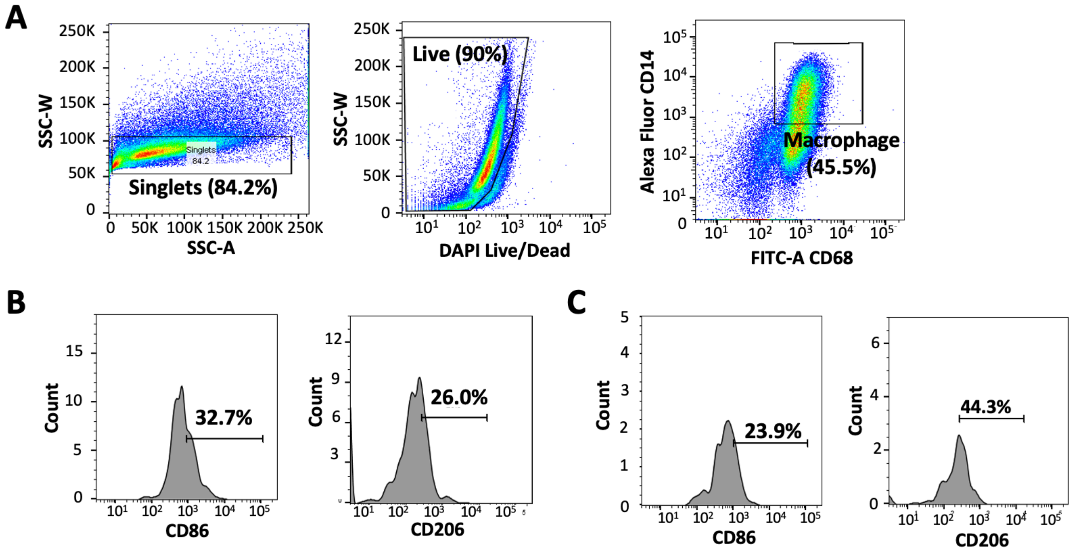

Supplementary Fig. S2. The gating strategy and phenotype analyses of THP1-derived macrophages upon 3-days stimulation with HKTB. (A) Gating strategy in the flow cytometry analysis for isolation of CD14^{+}CD68^{+} macrophages. The percentage of CD86^{+} (M1 phenotype) and CD206^{+} (M2 phenotype) macrophages in the (B) M and (C) TEM models.

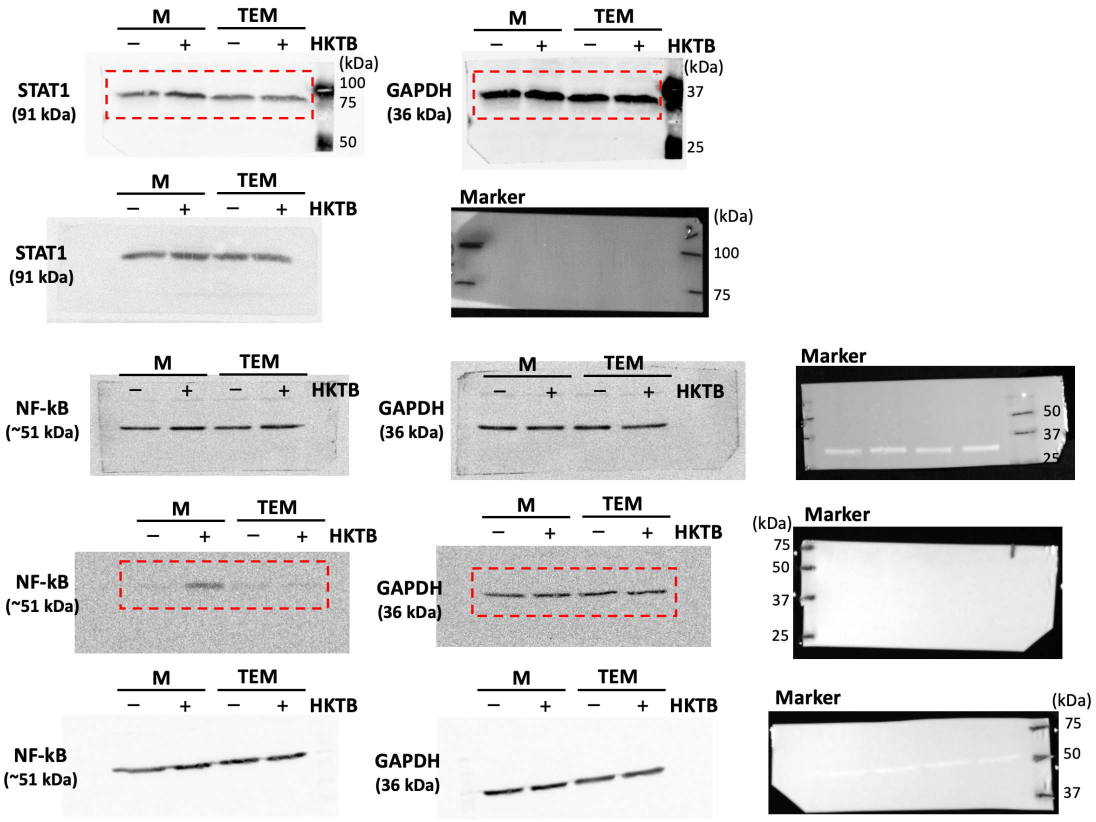

Supplementary Fig. S3. The images of western blot experiments. The images and PVDF membranes for western blotting analysis of STAT1, NF-κB and the corresponding loading control GAPDH were presented. The red dash-line rectangles indicate the images used in **Fig. 4A**.

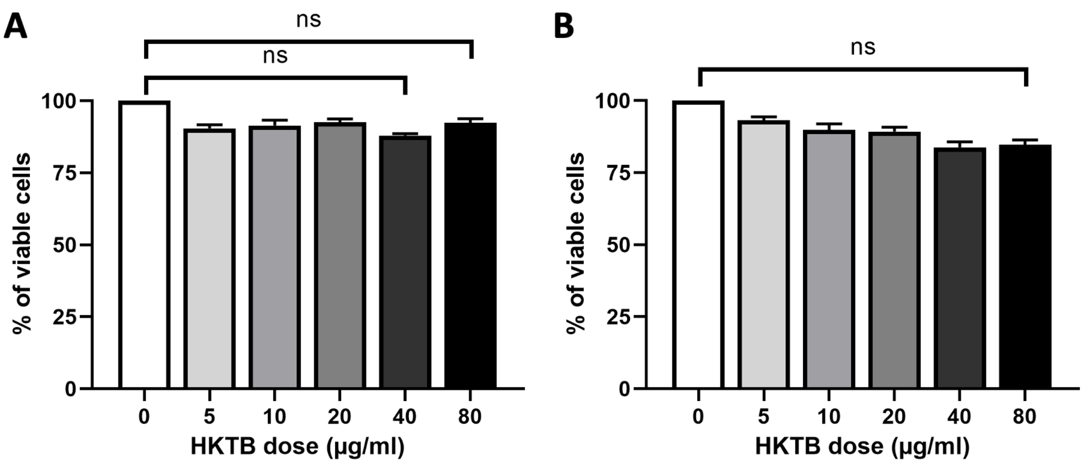

Supplementary Fig. S4. Viability analysis of A549 cells and THP1-derived macrophages. MTT assay was applied to determine the cell viability of (A) A549 cells and (B) THP1-derived macrophages upon direct stimulation with different doses of HKTB. Statistical analyses were performed by one-way ANOVA with Dunnett's multiple comparisons test. ns: not significant.

Supplementary Table S1. Subject characteristics

Characteristics	Healthy Subjects (n= 4)	Lung cancer (n=6)
**Age (n, %)**		
<40	2 (50)	0
40-59	2 (50)	5 (83.33)
≥60	0	1 (16.67)
**Gender (n, %)**		
Male	3 (75)	2 (33.33)
Female	1 (25)	4 (66.67)
**Diagnosis (n, %)**		
Adenocarcinoma	-	5 (83.33)
Squamous cell carcinoma	-	1 (16.67)
**Tumor stage (n, %)**		
Stage IV	-	6 (100)
**Mutation status (n, %)**		
EGFR L858R mutation	-	2 (33.33)
EGFR exon 19 deletion	-	1 (16.67)
EGFR wildtype	-	2 (33.33)
ALK mutation	-	1 (16.67)
**White blood cell count (n, %)**		
Within normal range	4 (100)	3 (50)
Leukocytosis	-	2 (33.33)
Leucopenia	-	1 (16.67)

Supplementary Table S2. Differentially expressed proteins in TB-M group.

Accession	Protein Name	Gene Name	# Peptides	# PSMs	# Protein Unique Peptides	# Unique Peptides	M (TMT127)	MTB (TMT128)	Log2 Ratio (MTB/M)
P04114	Apolipoprotein B-100	APOB	3	5	2	2	13.5	109.4	↑ 2.909
P01584	Interleukin-1 beta	IL1B	1	1	1	1	179	1236.8	↑ 2.679
P02787	Serotransferrin	TF	1	8	1	1	159.6	1097.8	↑ 2.673
P08697	Alpha-2-antiplasmin	SERPINF2	1	2	1	1	178.3	1116.5	↑ 2.537
P02647	Apolipoprotein A-I	APOA1	1	10	1	1	50.6	304.3	↑ 2.479
Q494V2	Cilia- and flagella-associated protein 100	CFAP100	1	11	1	1	157.4	896.9	↑ 2.401
O95817	BAG family molecular chaperone regulator 3	BAG3	1	2	1	1	447.1	2525.9	↑ 2.389
P35670	Copper-transporting ATPase 2	ATP7B	1	6	1	1	174.4	971.7	↑ 2.369
P69905	Hemoglobin subunit alpha	HBA1	4	37	3	4	179.7	968.1	↑ 2.320
P04179	Superoxide dismutase [Mn], mitochondrial	SOD2	7	32	7	7	203.3	1085.5	↑ 2.308
P04004	Vitronectin	VTN	1	5	1	1	66.7	354.7	↑ 2.302
P02771	Alpha-fetoprotein	AFP	3	26	3	3	419.3	2154.3	↑ 2.252
Q9NZR1	Tropomodulin-2	TMOD2	2	6	1	1	198.4	942.9	↑ 2.140
P0C7P3	Protein SLFN14	SLFN14	1	1	1	1	198.2	911.4	↑ 2.092
A6NK02	Putative tripartite motif-containing protein 75	TRIM75P	1	1	1	1	191.8	788.2	↑ 1.930
Q9Y6V0	Protein piccolo	PCLO	1	13	1	1	67.4	271.7	↑ 1.902
P19823	Inter-alpha-trypsin inhibitor heavy chain H2	ITIH2	9	36	9	9	624	2461.4	↑ 1.871
Q9NUP9	Protein lin-7 homolog C	LIN7C	1	3	1	1	177.4	691.1	↑ 1.853
P43155	Carnitine O-acetyltransferase	CRAT	1	1	1	1	251.3	975.5	↑ 1.848
P01008	Antithrombin-III	SERPINC1	4	7	4	4	135.4	491.8	↑ 1.752
P02774	Vitamin D-binding protein	GC	1	1	1	1	255.8	917	↑ 1.733
Q13416	Origin recognition complex subunit 2	ORC2	3	5	3	3	205.3	732.1	↑ 1.725
P43652	Afamin	AFM	1	2	1	1	160.6	567.6	↑ 1.712
P01024	Complement C3	C3	6	14	6	6	306.7	1066	↑ 1.688
P20592	Interferon-induced GTP-binding protein Mx2	MX2	7	13	5	5	388.1	1345.6	↑ 1.685
P05161	Ubiquitin-like protein ISG15	ISG15	5	16	5	5	247.9	859.1	↑ 1.684
P35244	Replication protein A 14 kDa subunit	RPA3	1	1	1	1	56.2	192.9	↑ 1.670
P02768	Serum albumin	ALB	8	196	8	8	2845.1	8992.8	↑ 1.551
P02788	Lactotransferrin	LTF	2	7	2	2	230	721.9	↑ 1.541
P04217	Alpha-1B-glycoprotein	A1BG	1	2	1	1	164.9	514.2	↑ 1.532

P14598	Neutrophil cytosol factor 1	NCF1	14	42	0	14	795.9	2243.3	1.386
Q96PZ2	Protein FAM111A	FAM111A	1	1	1	1	518.2	1424.2	1.349
P02649	Apolipoprotein E	APOE	4	10	4	4	338.7	918.7	1.330
P20591	Interferon-induced GTP-binding protein Mx1	MX1	17	34	14	15	325.1	879	1.326
Q14624	Inter-alpha-trypsin inhibitor heavy chain H4	ITIH4	2	4	2	2	181.7	490.8	1.324
P30613	Pyruvate kinase PKLR	PKLR	2	22	1	1	303.9	793.5	1.275
Q03181	Peroxisome proliferator-activated receptor delta	PPARD	1	2	1	1	443.7	1157.7	1.274
Q16587	Zinc finger protein 74	ZNF74	1	1	1	1	487	1262.8	1.265
O15479	Melanoma-associated antigen B2	MAGEB2	1	2	1	1	132.4	332.7	1.220
Q06033	Inter-alpha-trypsin inhibitor heavy chain H3	ITIH3	2	4	2	2	176.2	441.2	1.215
P02749	Beta-2-glycoprotein 1	APOH	1	4	1	1	35.8	89.6	1.214
P09914	Interferon-induced protein with tetratricopeptide repeats 1	IFIT1	3	7	3	3	446.7	1114.5	1.210
Q9H0R5	Guanylate-binding protein 3	GBP3	1	2	0	1	493.6	1227.6	1.205
P09601	Heme oxygenase 1	HMOX1	11	55	11	11	384.2	872.1	1.073
Q9NRL2	Bromodomain adjacent to zinc finger domain protein 1A	BAZ1A	2	2	2	2	583.1	1319.5	1.069
Q9Y6K5	2'-5'-oligoadenylate synthase 3	OAS3	11	20	11	11	343.3	775.3	1.066
Q8N9H6	Putative uncharacterized protein C8orf31	C8orf31	1	1	1	1	304	673.8	1.039
P29966	Myristoylated alanine-rich C-kinase substrate	MARCKS	5	6	4	5	144.9	319.6	1.032
Q9C0D4	Zinc finger protein 518B	ZNF518B	1	2	1	1	105	227.4	1.006
P07359	Platelet glycoprotein Ib alpha chain	GP1BA	1	1	1	1	338.5	725.2	0.990
O95786	Probable ATP-dependent RNA helicase DDX58	DDX58	1	1	1	1	689.2	1473.3	0.987
P19827	Inter-alpha-trypsin inhibitor heavy chain H1	ITIH1	1	2	1	1	273.8	583.9	0.983
Q9Y3U8	60S ribosomal protein L36	RPL36	2	3	2	2	99.2	211.3	0.982
Q92575	UBX domain-containing protein 4	UBXN4	1	2	1	1	456.3	961.5	0.966
P32456	Guanylate-binding protein 2	GBP2	1	1	1	1	721.2	1513	0.960
Q96G23	Ceramide synthase 2	CERS2	3	5	3	3	89.6	185.2	0.938
P29353	SHC-transforming protein 1	SHC1	1	1	0	1	64	131.9	0.934
P01042	Kininogen-1	KNG1	1	2	1	1	91.9	188.1	0.924
P49747	Cartilage oligomeric matrix protein	COMP	1	2	1	1	294.7	602.9	0.924
Q8IY81	pre-rRNA processing protein FTSJ3	FTSJ3	2	2	2	2	35.2	71.1	0.905
Q86UK7	E3 ubiquitin-protein ligase ZNF598	ZNF598	1	1	1	1	322.6	650.6	0.903
O14879	Interferon-induced protein with tetratricopeptide repeats 3	IFIT3	6	9	6	6	420	846.6	0.902
P50579	Methionine aminopeptidase 2	METAP2	1	1	1	1	69.4	138.1	0.884
Q9H3H5	UDP-N-acetylglucosamine--dolichyl-phosphate N-acetylglucosaminephosphotransferase	DPAGT1	1	3	1	1	64	126.2	0.870

Q8WVM8	Sec1 family domain-containing protein 1	SCFD1	5	8	5	5	462.9	907.4	↑ 0.862
P29590	Protein PML	PML	8	16	8	8	323.9	632.7	↑ 0.857
Q30154	HLA class II histocompatibility antigen, DR beta 5 chain	HLA-DRB5	4	14	0	3	245.7	477.8	↑ 0.850
P50583	Bis(5'-nucleosyl)-tetraphosphatase [asymmetrical]	NUDT2	1	2	1	1	158.3	304.4	↑ 0.834
P01911	HLA class II histocompatibility antigen, DRB1-15 beta chain	HLA-DRB1	4	15	0	3	461.8	873.4	↑ 0.810
P42224	Signal transducer and activator of transcription 1-alpha/beta	STAT1	19	45	19	19	272.3	514.7	↑ 0.809
P01892	HLA class I histocompatibility antigen, A-2 alpha chain	HLA-A	8	36	0	3	193.2	364.4	↑ 0.806
Q9HB58	Sp110 nuclear body protein	SP110	2	2	2	2	328	618.4	↑ 0.806
P01023	Alpha-2-macroglobulin	A2M	9	32	7	9	852	1603.1	↑ 0.803
P00395	Cytochrome c oxidase subunit 1	MT-CO1	1	2	1	1	74.4	139.8	↑ 0.801
P00973	2'-5'-oligoadenylate synthase 1	OAS1	1	2	1	1	105.8	197.4	↑ 0.791
Q92947	Glutaryl-CoA dehydrogenase, mitochondrial	GCDH	1	1	1	1	446.4	828.3	↑ 0.783
P20339	Ras-related protein Rab-5A	RAB5A	6	19	4	4	321	592.8	↑ 0.776
Q01628	Interferon-induced transmembrane protein 3	IFITM3	1	1	0	1	91.7	168.6	↑ 0.769
O14976	Cyclin-G-associated kinase	GAK	2	3	2	2	323.9	588.9	↑ 0.753
Q16831	Uridine phosphorylase 1	UPP1	2	3	2	2	180.5	327.3	↑ 0.749
P12277	Creatine kinase B-type	CKB	5	8	5	5	93.1	167.2	↑ 0.736
P01903	HLA class II histocompatibility antigen, DR alpha chain	HLA-DRA	3	9	2	3	75.2	134.9	↑ 0.734
P14780	Matrix metalloproteinase-9	MMP9	2	2	2	2	427.4	765.8	↑ 0.732
P23142	Fibulin-1	FBLN1	3	9	3	3	106.4	190.6	↑ 0.732
P35555	Fibrillin-1	FBN1	1	2	1	1	193.9	347	↑ 0.730
P25774	Cathepsin S	CTSS	3	6	3	3	67.5	120.3	↑ 0.725
P07858	Cathepsin B	CTSB	2	5	2	2	321.5	568.6	↑ 0.713
Q96T37	RNA-binding protein 15	RBM15	2	5	2	2	69.4	121.9	↑ 0.704
P0C0L4	Complement C4-A	C4A	5	9	0	5	675.9	1179.9	↑ 0.695
P04233	HLA class II histocompatibility antigen gamma chain	CD74	6	12	6	6	186.7	323.9	↑ 0.686
P05362	Intercellular adhesion molecule 1	ICAM1	8	17	8	8	229.4	396.3	↑ 0.680
P30479	HLA class I histocompatibility antigen, B-41 alpha chain	HLA-B	5	18	0	1	393.6	667.7	↑ 0.653
P29218	Inositol monophosphatase 1	IMPA1	1	1	1	1	324.3	549.2	↑ 0.651
O60603	Toll-like receptor 2	TLR2	2	3	2	2	63.3	107	↑ 0.648
Q5JRX3	Presequence protease, mitochondrial	PITRM1	5	5	5	5	160	268.6	↑ 0.638
Q02318	Sterol 26-hydroxylase, mitochondrial	CYP27A1	1	1	1	1	494.5	823.8	↑ 0.627
Q9BX68	Histidine triad nucleotide-binding protein 2, mitochondrial	HINT2	1	1	1	1	52.4	86.5	↑ 0.614
O43633	Charged multivesicular body protein 2a	CHMP2A	2	2	2	2	207.4	341.5	↑ 0.610
Q9BUL8	Programmed cell death protein 10	PDCD10	1	2	1	1	193	317.2	↑ 0.608

P21730	C5a anaphylatoxin chemotactic receptor 1	C5AR1	1	1	1	1	525.3	857.4	↑ 0.598
P61916	NPC intracellular cholesterol transporter 2	NPC2	2	3	2	2	270.5	441.1	↑ 0.596
Q9BT23	LIM domain-containing protein 2	LIMD2	1	2	1	1	481	783.8	↑ 0.595
P07947	Tyrosine-protein kinase Yes	YES1	4	10	0	1	388.3	628.6	↑ 0.586
P30273	High affinity immunoglobulin epsilon receptor subunit gamma	FCER1G	3	18	3	3	777.2	1256.1	↑ 0.583
Q7Z4Q2	HEAT repeat-containing protein 3	HEATR3	2	2	2	2	97.5	157.4	↑ 0.582
O43324	Eukaryotic translation elongation factor 1 epsilon-1	EEF1E1	2	2	2	2	185.8	299.9	↑ 0.582
Q9P2X0	Dolichol-phosphate mannosyltransferase subunit 3	DPM3	1	1	1	1	510.3	819.9	↑ 0.575
P30405	Peptidyl-prolyl cis-trans isomerase F, mitochondrial	PPIF	5	18	3	3	545.4	866.8	↑ 0.559
Q9NP72	Ras-related protein Rab-18	RAB18	3	3	3	3	130.6	207.5	↑ 0.559
Q969V3	Nicalin	NCLN	4	5	4	4	76.4	121.2	↑ 0.557
Q96EQ0	Small glutamine-rich tetratricopeptide repeat-containing protein beta	SGTB	1	1	1	1	414.7	657	↑ 0.555
O43914	TYRO protein tyrosine kinase-binding protein	TYROBP	2	5	2	2	346.2	548.2	↑ 0.554
Q9UJ68	Mitochondrial peptide methionine sulfoxide reductase	MSRA	4	7	4	4	49	77.5	↑ 0.552
P02042	Hemoglobin subunit delta	HBD	2	11	0	2	64.9	102.2	↑ 0.546
Q460N5	Protein mono-ADP-ribosyltransferase PARP14	PARP14	1	1	1	1	737.8	1160.9	↑ 0.545
Q10570	Cleavage and polyadenylation specificity factor subunit 1	CPSF1	2	3	2	2	87.9	138.2	↑ 0.544
Q9HCD5	Nuclear receptor coactivator 5	NCOA5	1	1	1	1	583.2	912.5	↑ 0.537
Q8N2K0	Monoacylglycerol lipase ABHD12	ABHD12	2	2	2	2	275.4	427.7	↑ 0.526
P07602	Prosaposin	PSAP	4	11	4	4	389.3	604.3	↑ 0.525
P14678	Small nuclear ribonucleoprotein-associated proteins B and B'	SNRPB	4	15	0	4	86.2	133.8	↑ 0.525
Q03519	Antigen peptide transporter 2	TAP2	6	12	6	6	535.4	830.6	↑ 0.524
P08631	Tyrosine-protein kinase HCK	HCK	4	10	1	1	49.4	76.3	↑ 0.518
Q14956	Transmembrane glycoprotein NMB	GPNMB	1	2	1	1	305.7	469.6	↑ 0.510
Q6I9Y2	THO complex subunit 7 homolog	THOC7	1	1	1	1	28.5	43.7	↑ 0.508
Q9NSU2	Three-prime repair exonuclease 1	TREX1	1	1	1	1	532	812.9	↑ 0.503
Q92609	TBC1 domain family member 5	TBC1D5	2	4	2	2	363.8	555.6	↑ 0.502
P42768	Wiskott-Aldrich syndrome protein	WAS	1	2	1	1	26.4	40.3	↑ 0.501
P04080	Cystatin-B	CSTB	2	9	2	2	473.9	721.4	↑ 0.497
P62714	Serine/threonine-protein phosphatase 2A catalytic subunit beta isoform	PPP2CB	5	11	1	1	439.3	668.5	↑ 0.497
O15027	Protein transport protein Sec16A	SEC16A	4	5	4	4	534.1	812.4	↑ 0.496
P11172	Uridine 5'-monophosphate synthase	UMPS	2	2	2	2	56.7	86.1	↑ 0.494
P28838	Cytosol aminopeptidase	LAP3	20	52	20	20	1096.2	1663.8	↑ 0.493

P62312	U6 snRNA-associated Sm-like protein LSm6	LSM6	3	4	3	3	319.2	484	↑ 0.491
P54920	Alpha-soluble NSF attachment protein	NAPA	6	11	5	6	889.6	1345.5	↑ 0.488
P09467	Fructose-1,6-bisphosphatase 1	FBP1	1	1	1	1	58	87.7	↑ 0.487
Q14554	Protein disulfide-isomerase A5	PDIA5	1	1	1	1	192.7	291	↑ 0.486
Q13287	N-myc-interactor	NMI	2	2	2	2	360.8	544.4	↑ 0.484
P23381	Tryptophan--tRNA ligase, cytoplasmic	WARS	5	8	5	5	361.7	544.7	↑ 0.482
Q15477	Helicase SKI2W	SKIV2L	2	2	2	2	98.6	148.4	↑ 0.481
P29466	Caspase-1	CASP1	2	2	2	2	665.4	999.3	↑ 0.478
Q02818	Nucleobindin-1	NUCB1	14	26	14	14	307.4	460.6	↑ 0.474
O75792	Ribonuclease H2 subunit A	RNASEH2A	2	3	2	2	72.8	109	↑ 0.473
P27635	60S ribosomal protein L10	RPL10	2	17	2	2	245.2	366.6	↑ 0.471
Q9H4M9	EH domain-containing protein 1	EHD1	7	10	2	5	143.3	214.2	↑ 0.471
Q00653	Nuclear factor NF-kappa-B p100 subunit	NFKB2	1	1	1	1	107.1	159.9	↑ 0.469
Q96HS1	Serine/threonine-protein phosphatase PGAM5, mitochondrial	PGAM5	3	6	3	3	211.6	315.3	↑ 0.466
Q14699	Raftlin	RFTN1	3	5	3	3	336.4	501.2	↑ 0.466
Q9Y4P3	Transducin beta-like protein 2	TBL2	2	3	2	2	235.7	350.7	↑ 0.464
Q8NEJ9	Neuroguidin	NGDN	1	1	1	1	59.5	88.2	↑ 0.459
P10319	HLA class I histocompatibility antigen, B-58 alpha chain	HLA-B	5	21	0	1	131.9	195.4	↑ 0.458
O43747	AP-1 complex subunit gamma-1	AP1G1	7	11	7	7	131.6	194.7	↑ 0.456
Q16352	Alpha-internexin	INA	17	53	13	16	539	797.1	↑ 0.455
Q9NTX5	Ethylmalonyl-CoA decarboxylase	ECHDC1	6	7	6	6	313.2	463.1	↑ 0.455
P12236	ADP/ATP translocase 3	SLC25A6	13	66	2	3	277.1	409.4	↑ 0.454
Q02224	Centromere-associated protein E	CENPE	1	1	1	1	823.5	1216.5	↑ 0.454
P43490	Nicotinamide phosphoribosyltransferase	NAMPT	5	9	5	5	126.8	187.2	↑ 0.453
O15347	High mobility group protein B3	HMGB3	3	6	3	3	186.6	275.2	↑ 0.451
O60739	Eukaryotic translation initiation factor 1b	EIF1B	3	6	1	3	49.3	72.7	↑ 0.451
Q14197	Peptidyl-tRNA hydrolase ICT1, mitochondrial	MRPL58	1	2	1	1	70.9	104.5	↑ 0.451
P42765	3-ketoacyl-CoA thiolase, mitochondrial	ACAA2	8	17	8	8	326.4	480	↑ 0.447
Q96L93	Kinesin-like protein KIF16B	KIF16B	1	2	0	1	67.2	98.8	↑ 0.447
O95456	Proteasome assembly chaperone 1	PSMG1	1	2	1	1	152.6	223.9	↑ 0.444
P80217	Interferon-induced 35 kDa protein	IFI35	2	3	2	2	462.4	677.9	↑ 0.443
O60488	Long-chain-fatty-acid--CoA ligase 4	ACSL4	2	2	2	2	141.6	207.3	↑ 0.441
Q03518	Antigen peptide transporter 1	TAP1	6	20	6	6	301.3	440.1	↑ 0.437
Q5T8P6	RNA-binding protein 26	RBM26	1	1	1	1	101.1	147.4	↑ 0.435
P19971	Thymidine phosphorylase	TYMP	15	48	15	15	768.2	1119.6	↑ 0.434

Q9H1C4	Protein unc-93 homolog B1	UNC93B1	2	3	2	2	666.9	970	⇧ 0.431
Q96PP9	Guanylate-binding protein 4	GBP4	1	1	1	1	404.5	585.4	⇧ 0.424
Q8N766	ER membrane protein complex subunit 1	EMC1	3	5	3	3	424.9	614.6	⇧ 0.423
Q8NF50	Dedicator of cytokinesis protein 8	DOCK8	10	19	10	10	215.2	311	⇧ 0.422
Q9UIG0	Tyrosine-protein kinase BAZ1B	BAZ1B	4	4	4	4	284.9	411.5	⇧ 0.421
Q9UIA9	Exportin-7	XPO7	1	2	1	1	68.1	98.2	⇧ 0.419
Q9NZ01	Very-long-chain enoyl-CoA reductase	TECR	7	18	7	7	201.9	291	⇧ 0.418
Q96T76	MMS19 nucleotide excision repair protein homolog	MMS19	2	3	2	2	109.5	156.6	⇧ 0.407
Q13501	Sequestosome-1	SQSTM1	2	2	2	2	56.5	80.8	⇧ 0.407
P12109	Collagen alpha-1(VI) chain	COL6A1	10	19	10	10	242.6	346.7	⇧ 0.406
Q53EP0	Fibronectin type III domain-containing protein 3B	FNDC3B	1	1	1	1	33.4	47.7	⇧ 0.405
A1L168	Uncharacterized protein C20orf202	C20orf202	1	1	1	1	266.7	379.9	⇧ 0.401
Q9BZL1	Ubiquitin-like protein 5	UBL5	1	1	1	1	610.7	869.4	⇧ 0.400
O95757	Heat shock 70 kDa protein 4L	HSPA4L	3	9	1	1	66.1	94.1	⇧ 0.400
P16070	CD44 antigen	CD44	5	16	5	5	567.3	807.6	⇧ 0.400
Q96A35	39S ribosomal protein L24, mitochondrial	MRPL24	1	2	1	1	339.5	483.2	⇧ 0.400
Q16719	Kynureninase	KYNU	3	3	3	3	66	93.9	⇧ 0.400
P42331	Rho GTPase-activating protein 25	ARHGAP25	2	2	2	2	42.4	60.1	⇧ 0.394
Q5H9U9	Probable ATP-dependent RNA helicase DDX60-like	DDX60L	1	1	1	1	72.5	102.6	⇧ 0.392
P17813	Endoglin	ENG	4	5	4	4	231.4	327.3	⇧ 0.391
P49459	Ubiquitin-conjugating enzyme E2 A	UBE2A	1	1	1	1	420.3	593.7	⇧ 0.389
P56378	6.8 kDa mitochondrial proteolipid	MP68	2	6	2	2	195.8	276.2	⇧ 0.387
Q8TD55	Pleckstrin homology domain-containing family O member 2	PLEKHO2	6	8	6	6	319.3	450.2	⇧ 0.387
Q69YN4	Protein virilizer homolog	VIRMA	1	2	1	1	630.5	888.8	⇧ 0.386
Q96Q15	Serine/threonine-protein kinase SMG1	SMG1	1	1	1	1	33	46.5	⇧ 0.386
Q9NSY1	BMP-2-inducible protein kinase	BMP2K	1	1	1	1	445.1	626.7	⇧ 0.385
P54725	UV excision repair protein RAD23 homolog A	RAD23A	5	14	3	3	381.7	537.4	⇧ 0.384
Q8TBM8	DnaJ homolog subfamily B member 14	DNAJB14	1	1	1	1	344.4	484.8	⇧ 0.384
Q9Y2B0	Protein canopy homolog 2	CNPY2	2	3	2	2	64.8	91.2	⇧ 0.384
Q63HN8	E3 ubiquitin-protein ligase RNF213	RNF213	5	7	5	5	458	643.8	⇧ 0.382
Q15021	Condensin complex subunit 1	NCAPD2	4	4	4	4	627.5	882	⇧ 0.382
P33121	Long-chain-fatty-acid--CoA ligase 1	ACSL1	12	27	10	12	455.1	639.3	⇧ 0.381
Q99590	Protein SCAF11	SCAF11	5	6	5	5	743.1	615.7	⇩ -0.380
P07195	L-lactate dehydrogenase B chain	LDHB	14	110	12	12	2291.5	1892.9	⇩ -0.385
Q13310	Polyadenylate-binding protein 4	PABPC4	16	45	9	11	694.6	572.8	⇩ -0.387

P12268	Inosine-5'-monophosphate dehydrogenase 2	IMPDH2	15	58	13	15	1584.3	1305.5	-0.388
Q9HB71	Calcyclin-binding protein	CACYBP	7	33	7	7	1206.9	993.3	-0.390
O95373	Importin-7	IPO7	12	27	12	12	647.4	532.7	-0.390
P40925	Malate dehydrogenase, cytoplasmic	MDH1	12	69	12	12	2472	2030	-0.393
P16930	Fumarylacetoacetase	FAH	3	9	3	3	715.1	587	-0.394
P20701	Integrin alpha-L	ITGAL	13	25	13	13	744.2	608.8	-0.399
Q86VM9	Zinc finger CCCH domain-containing protein 18	ZC3H18	2	3	2	2	1262	1031.9	-0.400
P35659	Protein DEK	DEK	4	11	4	4	976.5	798.4	-0.400
Q9UPN7	Serine/threonine-protein phosphatase 6 regulatory subunit 1	PPP6R1	1	2	1	1	542.8	443.7	-0.400
P07951	Tropomyosin beta chain	TPM2	11	42	1	1	1012.3	826.7	-0.401
P33991	DNA replication licensing factor MCM4	MCM4	18	45	17	18	1703.8	1390.2	-0.403
O00267	Transcription elongation factor SPT5	SUPT5H	4	6	4	4	451.7	368	-0.405
Q99733	Nucleosome assembly protein 1-like 4	NAP1L4	7	18	6	6	1010.2	822.6	-0.406
Q14444	Caprin-1	CAPRIN1	11	32	11	11	1285.6	1046.2	-0.406
Q8NBS9	Thioredoxin domain-containing protein 5	TXNDC5	2	3	2	2	58.2	47.3	-0.408
O43175	D-3-phosphoglycerate dehydrogenase	PHGDH	6	12	6	6	403.7	327.8	-0.410
Q9NX14	NADH dehydrogenase [ubiquinone] 1 beta subcomplex subunit 11, mitochondrial	NDUFB11	2	2	2	2	658.1	533.1	-0.413
P52292	Importin subunit alpha-1	KPNA2	5	10	5	5	425.3	344.5	-0.413
P13489	Ribonuclease inhibitor	RNH1	6	30	6	6	707.7	571.8	-0.417
Q9NV96	Cell cycle control protein 50A	TMEM30A	2	2	2	2	183.4	148.1	-0.418
O95936	Eomesodermin homolog	EOMES	1	2	1	1	983.6	794.1	-0.418
O75821	Eukaryotic translation initiation factor 3 subunit G	EIF3G	3	8	3	3	563.1	453.7	-0.421
P06276	Cholinesterase	BCHE	1	3	1	1	1147.6	924	-0.422
P60709	Actin, cytoplasmic 1	ACTB	18	861	1	1	348.6	279.7	-0.427
Q9HAV4	Exportin-5	XPO5	6	9	6	6	1077.6	863.5	-0.429
Q6PKG0	La-related protein 1	LARP1	10	18	10	10	715.2	570.2	-0.436
Q9BV40	Vesicle-associated membrane protein 8	VAMP8	4	16	4	4	507.6	404.5	-0.437
Q9GZZ1	N-alpha-acetyltransferase 50	NAA50	2	3	2	2	240.9	191.1	-0.443
P13647	Keratin, type II cytoskeletal 5	KRT5	32	190	17	17	3247.7	2574.6	-0.444
Q86VI3	Ras GTPase-activating-like protein IQGAP3	IQGAP3	4	11	2	2	546	431.8	-0.448
P33316	Deoxyuridine 5'-triphosphate nucleotidohydrolase, mitochondrial	DUT	4	10	4	4	596.6	471.3	-0.449
Q58FF6	Putative heat shock protein HSP 90-beta 4	HSP90AB4P	5	39	1	1	670.9	529.6	-0.450
A4D1E9	GTP-binding protein 10	GTPBP10	1	1	1	1	150.7	118	-0.462
Q9BTE3	Mini-chromosome maintenance complex-binding protein	MCMBP	1	2	1	1	759.5	594.4	-0.463

O60496	Docking protein 2	DOK2	2	2	2	2	572.1	444.3	-0.474
Q5T3U5	Multidrug resistance-associated protein 7	ABCC10	1	2	1	1	1414	1097.7	-0.474
O75369	Filamin-B	FLNB	37	94	29	37	2903.9	2252.8	-0.475
Q9UG63	ATP-binding cassette sub-family F member 2	ABCF2	7	8	7	7	455.3	350.8	-0.485
Q15185	Prostaglandin E synthase 3	PTGES3	3	11	3	3	542.5	416.9	-0.489
Q9Y5A9	YTH domain-containing family protein 2	YTHDF2	4	6	1	1	100.1	76.5	-0.497
Q12834	Cell division cycle protein 20 homolog	CDC20	1	2	1	1	1468.9	1118.6	-0.502
P11388	DNA topoisomerase 2-alpha	TOP2A	16	33	9	9	784.2	595.9	-0.505
O75475	PC4 and SFRS1-interacting protein	PSIP1	5	11	4	4	488.1	369.3	-0.512
P13645	Keratin, type I cytoskeletal 10	KRT10	40	711	30	32	12213.9	9237.3	-0.512
P13646	Keratin, type I cytoskeletal 13	KRT13	8	132	1	1	1391.8	1039.9	-0.530
O14777	Kinetochore protein NDC80 homolog	NDC80	1	1	1	1	678.2	500.8	-0.547
P24844	Myosin regulatory light polypeptide 9	MYL9	6	18	2	2	398.2	292.3	-0.555
Q96P63	Serpin B12	SERPINB12	2	3	2	2	796.6	582.9	-0.560
P61626	Lysozyme C	LYZ	2	5	2	2	1018	744.3	-0.561
P35908	Keratin, type II cytoskeletal 2 epidermal	KRT2	49	507	35	35	10554.5	7630.3	-0.577
O43432	Eukaryotic translation initiation factor 4 gamma 3	EIF4G3	4	7	1	1	74.9	53.7	-0.589
P02533	Keratin, type I cytoskeletal 14	KRT14	29	188	11	13	1237.3	878.6	-0.603
P04264	Keratin, type II cytoskeletal 1	KRT1	46	717	37	38	21318.8	15043.2	-0.612
P02538	Keratin, type II cytoskeletal 6A	KRT6A	26	155	1	2	694.8	484.1	-0.630
O60566	Mitotic checkpoint serine/threonine-protein kinase BUB1 beta	BUB1B	1	1	1	1	769	532.3	-0.640
Q86YZ3	Hornerin	HRNR	29	132	29	29	2956.6	2021.9	-0.657
Q9NUQ8	ATP-binding cassette sub-family F member 3	ABCF3	1	2	1	1	662	451.5	-0.661
P35527	Keratin, type I cytoskeletal 9	KRT9	34	352	33	33	10488.3	7055.5	-0.681
Q13835	Plakophilin-1	PKP1	1	1	1	1	383.6	233.9	-0.823
Q96KA5	Cleft lip and palate transmembrane protein 1-like protein	CLPTM1L	1	1	1	1	199.7	115.4	-0.900
Q8IX19	Mast cell-expressed membrane protein 1	MCEMP1	4	11	4	4	788.7	454.2	-0.905
P68431	Histone H3.1	HIST1H3A	5	95	0	1	888	436.4	-1.134

Supplementary Table S3. Differentially expressed proteins in TB-TEM group.

Accession	Description	Gene Name	# Peptides	# PSMs	# Protein Unique Peptides	# Unique Peptides	TEM (TMT129)	TTB (TMT130)	Log2 Ratio (TTB/TEM)
P01584	Interleukin-1 beta	IL1B	1	1	1	1	153.7	518	↑ 1.755
P04179	Superoxide dismutase [Mn], mitochondrial	SOD2	7	32	7	7	316.3	775.3	↑ 1.295
Q9NUP9	Protein lin-7 homolog C	LIN7C	1	3	1	1	164.7	402.2	↑ 1.290
A6NK02	Putative tripartite motif-containing protein 75	TRIM75P	1	1	1	1	239.6	514.6	↑ 1.105
Q14699	Raftlin	RFTN1	3	5	3	3	479.9	1002.3	↑ 1.064
P61626	Lysozyme C	LYZ	2	5	2	2	620.7	1190.1	↑ 0.941
Q02318	Sterol 26-hydroxylase, mitochondrial	CYP27A1	1	1	1	1	557	1064	↑ 0.936
P05362	Intercellular adhesion molecule 1	ICAM1	8	17	8	8	293.2	527.1	↑ 0.848
P29966	Myristoylated alanine-rich C-kinase substrate	MARCKS	5	6	4	5	143.8	257	↑ 0.840
Q9UH99	SUN domain-containing protein 2	SUN2	5	5	5	5	202.1	354.6	↑ 0.813
Q9NRL2	Bromodomain adjacent to zinc finger domain protein 1A	BAZ1A	2	2	2	2	601.7	1031.8	↑ 0.780
Q96RQ9	L-amino-acid oxidase	IL4I1	1	2	1	1	71.2	122	↑ 0.779
P30479	HLA class I histocompatibility antigen, B-41 alpha chain	HLA-B	5	18	0	1	483.3	827.9	↑ 0.778
P30405	Peptidyl-prolyl cis-trans isomerase F, mitochondrial	PPIF	5	18	3	3	598.6	998	↑ 0.739
P07359	Platelet glycoprotein Ib alpha chain	GP1BA	1	1	1	1	272.6	453.5	↑ 0.736
Q16587	Zinc finger protein 74	ZNF74	1	1	1	1	541.2	894.9	↑ 0.727
P15144	Aminopeptidase N	ANPEP	19	64	19	19	1381.9	2284.5	↑ 0.727
Q8N9H6	Putative uncharacterized protein C8orf31	C8orf31	1	1	1	1	304	499	↑ 0.717
P01892	HLA class I histocompatibility antigen, A-2 alpha chain	HLA-A	8	36	0	3	230.5	378.3	↑ 0.717
P17096	High mobility group protein HMG-I/HMG-Y	HMGA1	4	19	4	4	603.2	934.6	↑ 0.634
P07858	Cathepsin B	CTSB	2	5	2	2	482.8	740	↑ 0.618
P14598	Neutrophil cytosol factor 1	NCF1	14	42	0	14	762.4	1166.7	↑ 0.616
Q9HB58	Sp110 nuclear body protein	SP110	2	2	2	2	384.6	577.5	↑ 0.588
P51149	Ras-related protein Rab-7a	RAB7A	11	40	11	11	568.1	845.7	↑ 0.576
P02749	Beta-2-glycoprotein 1	APOH	1	4	1	1	58.4	85.8	↑ 0.557
Q92947	Glutaryl-CoA dehydrogenase, mitochondrial	GCDH	1	1	1	1	429.5	630.6	↑ 0.556
Q03519	Antigen peptide transporter 2	TAP2	6	12	6	6	605.2	886.1	↑ 0.552
Q15477	Helicase SKI2W	SKIV2L	2	2	2	2	97.2	141.2	↑ 0.541
P61916	NPC intracellular cholesterol transporter 2	NPC2	2	3	2	2	546.1	789.3	↑ 0.533

P15954	Cytochrome c oxidase subunit 7C, mitochondrial	COX7C	2	5	2	2	169.4	244	↑	0.528
P10319	HLA class I histocompatibility antigen, B-58 alpha chain	HLA-B	5	21	0	1	139.2	199.7	↑	0.523
Q7LGA3	Heparan sulfate 2-O-sulfotransferase 1	HS2ST1	1	1	1	1	161	230.8	↑	0.521
Q9BT23	LIM domain-containing protein 2	LIMD2	1	2	1	1	521.7	743.3	↑	0.513
P20592	Interferon-induced GTP-binding protein Mx2	MX2	7	13	5	5	461.6	653	↑	0.502
P08648	Integrin alpha-5	ITGA5	4	5	4	4	231.9	326.4	↑	0.495
P16070	CD44 antigen	CD44	5	16	5	5	558.7	781.6	↑	0.486
O94925	Glutaminase kidney isoform, mitochondrial	GLS	5	9	5	5	710	992.7	↑	0.485
Q9Y241	HIG1 domain family member 1A, mitochondrial	HIGD1A	1	3	1	1	140.9	196.9	↑	0.485
P02649	Apolipoprotein E	APOE	4	10	4	4	550.5	764.2	↑	0.475
P23219	Prostaglandin G/H synthase 1	PTGS1	3	7	2	3	326.7	453.2	↑	0.474
P12109	Collagen alpha-1(VI) chain	COL6A1	10	19	10	10	403.5	559.4	↑	0.473
Q14108	Lysosome membrane protein 2	SCARB2	4	12	4	4	433.5	598.5	↑	0.467
Q9BX68	Histidine triad nucleotide-binding protein 2, mitochondrial	HINT2	1	1	1	1	49.1	67.4	↑	0.459
P56385	ATP synthase subunit e, mitochondrial	ATP5ME	1	2	1	1	812.8	1108.6	↑	0.450
Q9Y6I9	Testis-expressed protein 264	TEX264	2	3	2	2	370	504.6	↑	0.450
Q9Y385	Ubiquitin-conjugating enzyme E2 J1	UBE2J1	3	6	3	3	737.6	1004.8	↑	0.448
Q07021	Complement component 1 Q subcomponent-binding protein, mitochondrial	C1QBP	4	8	4	4	128	174.2	↑	0.447
A6NC86	phospholipase A2 inhibitor and Ly6/PLAUR domain-containing protein	PINLYP	1	3	1	1	162.8	221	↑	0.443
P17900	Ganglioside GM2 activator	GM2A	1	2	1	1	442.8	600.8	↑	0.442
P19256	Lymphocyte function-associated antigen 3	CD58	1	2	1	1	623.5	845.3	↑	0.441
Q13501	Sequestosome-1	SQSTM1	2	2	2	2	53.1	71.9	↑	0.439
P09601	Heme oxygenase 1	HMOX1	11	55	11	11	536.5	726.2	↑	0.439
Q8NBI5	Solute carrier family 43 member 3	SLC43A3	2	5	2	2	862.7	1163.4	↑	0.433
Q9P2X0	Dolichol-phosphate mannosyltransferase subunit 3	DPM3	1	1	1	1	662.4	891.9	↑	0.431
P62995	Transformer-2 protein homolog beta	TRA2B	6	10	5	5	204.8	272.4	↑	0.413
Q9NX63	MICOS complex subunit MIC19	CHCHD3	7	23	7	7	413.4	549	↑	0.411
O76021	Ribosomal L1 domain-containing protein 1	RSL1D1	8	14	8	8	586.4	776.3	↑	0.407
P05107	Integrin beta-2	ITGB2	14	88	13	13	1330.6	1756.6	↑	0.403
P05204	Non-histone chromosomal protein HMG-17	HMGN2	3	23	3	3	958.5	1265.2	↑	0.402
Q02978	Mitochondrial 2-oxoglutarate/malate carrier protein	SLC25A11	2	6	2	2	684.5	901.8	↑	0.400
P15289	Arylsulfatase A	ARSA	2	2	1	2	578.2	761	↑	0.398

P31949	Protein S100-A11	S100A11	4	19	4	4	535.1	703.9	↑	0.397
P30049	ATP synthase subunit delta, mitochondrial	ATP5F1D	2	7	2	2	423.7	556.8	↑	0.396
P99999	Cytochrome c	CYCS	4	9	4	4	167.6	219.4	↑	0.390
P21730	C5a anaphylatoxin chemotactic receptor 1	C5AR1	1	1	1	1	569.7	742.9	↑	0.385
Q9Y4P3	Transducin beta-like protein 2	TBL2	2	3	2	2	301	392.4	↑	0.384
Q9NQ55	Suppressor of SWI4 1 homolog	PPAN	4	6	4	4	788.7	1027.7	↑	0.384
Q96DI7	U5 small nuclear ribonucleoprotein 40 kDa protein	SNRNP40	3	3	3	3	132.5	172.3	↑	0.381
P14780	Matrix metalloproteinase-9	MMP9	2	2	2	2	373	484.8	↑	0.380
P20701	Integrin alpha-L	ITGAL	13	25	13	13	718.2	933	↑	0.379
Q9NRX4	14 kDa phosphohistidine phosphatase	PHPT1	4	14	4	4	713.9	547.8	↓	-0.380
P62942	Peptidyl-prolyl cis-trans isomerase FKBP1A	FKBP1A	2	8	2	2	476.2	362	↓	-0.394
Q96T88	E3 ubiquitin-protein ligase UHRF1	UHRF1	4	7	4	4	301.1	228.5	↓	-0.396
Q15274	Nicotinate-nucleotide pyrophosphorylase [carboxylating]	QPRT	3	7	3	3	740.3	561.3	↓	-0.397
P60709	Actin, cytoplasmic 1	ACTB	18	861	1	1	314.9	238.5	↓	-0.399
Q8N8S7	Protein enabled homolog	ENAH	1	1	1	1	952	719.1	↓	-0.403
Q96T76	MMS19 nucleotide excision repair protein homolog	MMS19	2	3	2	2	137	103.3	↓	-0.405
P81605	Dermcidin	DCD	4	6	4	4	414.7	312.2	↓	-0.408
P29508	Serpin B3	SERPINB3	3	4	0	3	561.4	421.9	↓	-0.410
O14737	Programmed cell death protein 5	PDCD5	3	7	3	3	1035.5	778.1	↓	-0.410
Q13835	Plakophilin-1	PKP1	1	1	1	1	218.2	163.9	↓	-0.411
P07108	Acyl-CoA-binding protein	DBI	3	4	3	3	156.4	117.3	↓	-0.413
P01911	HLA class II histocompatibility antigen, DRB1-15 beta chain	HLA-DRB1	4	15	0	3	1040.8	780.3	↓	-0.414
Q92973	Transportin-1	TNPO1	17	28	13	17	821.9	615.7	↓	-0.415
Q9C0D4	Zinc finger protein 518B	ZNF518B	1	2	1	1	180.8	134.9	↓	-0.421
P33241	Lymphocyte-specific protein 1	LSP1	7	14	7	7	178.9	133.2	↓	-0.424
Q9NTX5	Ethylmalonyl-CoA decarboxylase	ECHDC1	6	7	6	6	435.5	324.1	↓	-0.424
P58546	Myotrophin	MTPN	2	4	2	2	472.1	350.4	↓	-0.428
Q15042	Rab3 GTPase-activating protein catalytic subunit	RAB3GAP1	3	4	3	3	92.7	68.8	↓	-0.428
Q06033	Inter-alpha-trypsin inhibitor heavy chain H3	ITIH3	2	4	2	2	354.5	262.1	↓	-0.434
O95340	Bifunctional 3'-phosphoadenosine 5'-phosphosulfate synthase 2	PAPSS2	3	4	2	2	630.9	466.3	↓	-0.434
O95456	Proteasome assembly chaperone 1	PSMG1	1	2	1	1	195.3	143.7	↓	-0.441
Q13442	28 kDa heat- and acid-stable phosphoprotein	PDAP1	1	1	1	1	736.9	542	↓	-0.441
Q58FF6	Putative heat shock protein HSP 90-beta 4	HSP90AB4P	5	39	1	1	582.3	424.8	↓	-0.453

P07477	Trypsin-1	PRSS1	3	46	1	3	397.8	289.8	-0.455
Q06187	Tyrosine-protein kinase BTK	BTK	4	4	4	4	306.7	222.5	-0.461
Q30154	HLA class II histocompatibility antigen, DR beta 5 chain	HLA-DRB5	4	14	0	3	622.3	451	-0.463
P27635	60S ribosomal protein L10	RPL10	2	17	2	2	363.8	263.4	-0.464
Q9BV20	Methylthioribose-1-phosphate isomerase	MRI1	1	1	1	1	73.3	52.8	-0.471
P02647	Apolipoprotein A-I	APOA1	1	10	1	1	204.9	147.5	-0.472
P19827	Inter-alpha-trypsin inhibitor heavy chain H1	ITIH1	1	2	1	1	515.2	369.5	-0.478
Q03181	Peroxisome proliferator-activated receptor delta	PPARD	1	2	1	1	697.5	498.2	-0.484
P15090	Fatty acid-binding protein, adipocyte	FABP4	5	35	4	5	1384.4	984.9	-0.489
O15232	Matrilin-3	MATN3	1	3	1	1	201.9	143.2	-0.494
O60496	Docking protein 2	DOK2	2	2	2	2	407.1	288.7	-0.494
O14976	Cyclin-G-associated kinase	GAK	2	3	2	2	491	347.8	-0.496
Q86VI3	Ras GTPase-activating-like protein IQGAP3	IQGAP3	4	11	2	2	427.3	300.6	-0.505
Q8IX19	Mast cell-expressed membrane protein 1	MCEMP1	4	11	4	4	709.5	497.6	-0.510
P49747	Cartilage oligomeric matrix protein	COMP	1	2	1	1	504.8	352.4	-0.517
P07951	Tropomyosin beta chain	TPM2	11	42	1	1	1106.5	771.6	-0.518
P01042	Kininogen-1	KNG1	1	2	1	1	185.3	129.1	-0.519
P68366	Tubulin alpha-4A chain	TUBA4A	14	107	2	2	438.1	300.9	-0.540
Q92575	UBX domain-containing protein 4	UBXN4	1	2	1	1	781.1	534.9	-0.544
P02788	Lactotransferrin	LTF	2	7	2	2	633.5	426.5	-0.569
P68431	Histone H3.1	HIST1H3A	5	95	0	1	632.3	417.8	-0.596
Q9NQZ5	StAR-related lipid transfer protein 7, mitochondrial	STARD7	1	1	1	1	1036.2	666	-0.636
O15479	Melanoma-associated antigen B2	MAGEB2	1	2	1	1	290	185.2	-0.645
P01024	Complement C3	C3	6	14	6	6	895.5	563.3	-0.667
Q96CX2	BTB/POZ domain-containing protein KCTD12	KCTD12	3	5	3	3	441	274.9	-0.680
P50579	Methionine aminopeptidase 2	METAP2	1	1	1	1	138.6	86.2	-0.683
P02768	Serum albumin	ALB	8	196	8	8	7579.2	4683.9	-0.692
P43155	Carnitine O-acetyltransferase	CRAT	1	1	1	1	763.4	470.5	-0.696
P50583	Bis(5'-nucleosyl)-tetraphosphatase [asymmetrical]	NUDT2	1	2	1	1	257.1	158.3	-0.698
P35555	Fibrillin-1	FBN1	1	2	1	1	354.1	217	-0.705
Q14624	Inter-alpha-trypsin inhibitor heavy chain H4	ITIH4	2	4	2	2	391.2	239.5	-0.706
Q9ULK4	Mediator of RNA polymerase II transcription subunit 23	MED23	1	1	1	1	96.6	58.8	-0.714
P02774	Vitamin D-binding protein	GC	1	1	1	1	580.7	350.6	-0.726

P30613	Pyruvate kinase PKLR	PKLR	2	22	1	1	646.3	388.5	⇩	-0.732
P01008	Antithrombin-III	SERPINC1	4	7	4	4	373.7	224.1	⇩	-0.736
Q9Y6V0	Protein piccolo	PCLO	1	13	1	1	190.4	112.3	⇩	-0.760
P35244	Replication protein A 14 kDa subunit	RPA3	1	1	1	1	146.7	84.9	⇩	-0.787
P04217	Alpha-1B-glycoprotein	A1BG	1	2	1	1	493.5	281.5	⇩	-0.808
P04114	Apolipoprotein B-100	APOB	3	5	2	2	75.9	42.2	⇩	-0.845
Q13416	Origin recognition complex subunit 2	ORC2	3	5	3	3	613	338.4	⇩	-0.855
P19823	Inter-alpha-trypsin inhibitor heavy chain H2	ITIH2	9	36	9	9	1994.9	1090.8	⇩	-0.869
P0C7P3	Protein SLFN14	SLFN14	1	1	1	1	834.7	415.6	⇩	-1.004
P08697	Alpha-2-antiplasmin	SERPINF2	1	2	1	1	882.7	428.5	⇩	-1.041
P02771	Alpha-fetoprotein	AFP	3	26	3	3	1804.7	871.2	⇩	-1.049
Q494V2	Cilia- and flagella-associated protein 100	CFAP100	1	11	1	1	726.6	322	⇩	-1.172
P02787	Serotransferrin	TF	1	8	1	1	943.1	414.3	⇩	-1.185

Supplementary Table S4. Enriched pathways in HKTB stimulated M and TEM.

Ingenuity Canonical Pathways	HKTB-stimulated M (MTB)				HKTB-stimulated TEM (TTB)			
	-log (p-value)	Ratio	z-score	Molecules	-log (p-value)	Ratio	z-score	Molecules
LXR/RXR Activation	14.4	0.149	2.828	KNG1,APOE,APOB,C3,APOH,VTN,NFKB2,A1BG,SERPINF2,C4A/C4B,ALB,LYZ,APOA1,TF,ITIH4,IL1B,GC,MMP9	15	0.124	-2.84	KNG1,APOE,C3,APOB,APOH,A1BG,SERPINF2,ALB,LYZ,APOA1,TF,ITIH4,IL1B,GC,MMP9
FXR/RXR Activation	14.1	0.143		KNG1,APOE,APOB,C3,CYP27A1,APOH,PKLR,VTN,A1BG,SERPINF2,C4A/C4B,ALB,APOA1,TF, ITIH4,IL1B, FBP1,GC	14.7	0.119		KNG1,APOE,ALB,APOA1,APOB,C3,TF,APOH,PKLR,CYP27A1,ITIH4,IL1B,GC,SERPINF2,A1BG
Acute Phase Response Signaling	9.41	0.0894	1.414	ITIH3,C3,APOH,NFKB2,SERPINF2,C4A/C4B,SHC1,HMOX1,ALB,APOA1,SOD2,TF,ITIH2,ITIH4, IL1B,A2M	8.93	0.067	0.447	HMOX1,ITIH3,ALB,SOD2,C3,APOA1,TF,APOH,ITIH2,ITIH4,IL1B,SERPINF2
Clathrin-mediated Endocytosis Signaling	3.74	0.0481		APOE,LYZ,RAB5A,ALB,APOB,APOA1,TF,ACTB,GAK,AP1G1	7.16	0.0529		ITGB2,APOE,LYZ,ALB,APOB,APOA1,TF,ACTB,GAK,RAB7A,ITGA5
Atherosclerosis Signaling	4.69	0.0703		APOE,LYZ,ALB,APOB,APOA1,ICAM1,IL1B,NFKB2,MMP9	7	0.0703		ITGB2,APOE,LYZ,ALB,APOB,APOA1,ICAM1,IL1B,MMP9
Caveolar-mediated Endocytosis Signaling	4.71	0.0986		FLNB,RAB5A,ALB,HLA-A,ACTB,HLA-B,ITGAL	6.56	0.0986		ITGB2,ALB,HLA-A,ACTB,HLA-B,ITGA5,ITGAL
Antigen Presentation Pathway	7.84	0.205		HLA-DRB1,HLA-A,HLA-B,HLA-DRA,CD74,TAP2,TAP1,HLA-DRB5	5.4	0.128		HLA-DRB1,HLA-A,HLA-B,TAP2,HLA-DRB5
Leukocyte Extravasation Signaling	1.88	0.0329	1.134	NCF1,ICAM1,WAS,ACTB,CD44,ITGAL,MMP9	5.15	0.0423	1.667	BTK,ITGB2,NCF1,ICAM1,ACTB,CD44,ITGA5,ITGAL,MMP9
Graft-versus-Host Disease Signaling	5.86	0.146		HLA-DRB1,HLA-A,HLA-B,HLA-DRA,FCER1G,IL1B,HLA-DRB5	4.94	0.104		HLA-DRB1,HLA-A,HLA-B,IL1B,HLA-DRB5
Crosstalk between Dendritic Cells and Natural Killer Cells	5.99	0.101		HLA-DRB1,HLA-A,TYROBP,ACTB,HLA-B,HLA-DRA,NFKB2,ITGAL,HLA-DRB5	4.73	0.0674		HLA-DRB1,HLA-A,ACTB,HLA-B,ITGAL,HLA-DRB5
Neuroinflammation Signaling Pathway	5.32	0.0479	3.742	ICAM1,HLA-A,TYROBP,HLA-B,NFKB2,TLR2,HMOX1,SOD2,HLA-DRB1,HLA-DRA,CASP1,IL1B,STAT1,HLA-DRB5,MMP9	4.61	0.0319	1.897	HMOX1,SOD2,HLA-DRB1,ICAM1,HLA-A,GLS,HLA-B,IL1B,MMP9,HLA-DRB5
Nur77 Signaling in T Lymphocytes	4.18	0.102		HLA-DRB1,HLA-A,HLA-B,HLA-DRA,FCER1G,HLA-DRB5	4.5	0.0847		HLA-DRB1,HLA-A,HLA-B,CYCS,HLA-DRB5
Phagosome Maturation	4.99	0.0676		RAB5A,HLA-DRB1,CTSS,HLA-A,HLA-B,HLA-DRA,CTSB,NAPA,TAP1,HLA-DRB5	4.43	0.0473		HLA-DRB1,HLA-A,HLA-B,RAB7A,CTSB,TUBA4A,HLA-DRB5
Type I Diabetes Mellitus Signaling	5.19	0.0811	2	HLA-DRB1,HLA-A,HLA-B,HLA-DRA,FCER1G,IL1B,NFKB2,STAT1,HLA-DRB5	4.19	0.0541		HLA-DRB1,HLA-A,HLA-B,IL1B,CYCS,HLA-DRB5
B Cell Development	4.22	0.139		HLA-DRB1,HLA-A,HLA-B,HLA-DRA,HLA-DRB5	4.16	0.111		HLA-DRB1,HLA-A,HLA-B,HLA-DRB5
Complement System	2.04	0.0811		C4A/C4B,C3,C5AR1	4.11	0.108		ITGB2,C3,C5AR1,C1QBP
Virus Entry via Endocytic Pathways	2.51	0.0496		FLNB,HLA-A,ACTB,HLA-B,ITGAL,AP1G1	3.98	0.0496		ITGB2,HLA-A,ACTB,HLA-B,ITGA5,ITGAL
Agranulocyte Adhesion and Diapedesis	1.59	0.0312		MYL9,ICAM1,C5AR1,ACTB,IL1B,MMP9	3.72	0.0365		ITGB2,ICAM1,C5AR1,ACTB,IL1B,ITGA5,MMP9

Th1 Pathway	2.93	0.0511	2.449	ICAM1,HLA-DRB1,HLA-A,HLA-B,HLA-DRA,STAT1,HLA-DRB5	3.68	0.0438	0	ITGB2,HLA-DRB1,ICAM1,HLA-A,HLA-B,HLA-DRB5
Dendritic Cell Maturation	5.43	0.0612	3.317	TLR2,HLA-DRB1,ICAM1,HLA-A,TYROBP,HLA-B,HLA-DRA,FCER1G,IL1B,NFKB2,STAT1,HLA-DRB5	3.67	0.0357	1.134	HLA-DRB1,ICAM1,HLA-A,HLA-B,CD58,IL1B,HLA-DRB5
Altered T Cell and B Cell Signaling in Rheumatoid Arthritis	5.95	0.1		TLR2,HLA-DRB1,HLA-A,HLA-B,HLA-DRA,FCER1G,IL1B,NFKB2,HLA-DRB5	3.63	0.0556		HLA-DRB1,HLA-A,HLA-B,IL1B,HLA-DRB5
Autoimmune Thyroid Disease Signaling	4.65	0.122		HLA-DRB1,HLA-A,HLA-B,HLA-DRA,FCER1G,HLA-DRB5	3.63	0.0816		HLA-DRB1,HLA-A,HLA-B,HLA-DRB5
IL-12 Signaling and Production in Macrophages	3.44	0.0541		TLR2,APOE,LYZ,ALB,APOA1,APOB,NFKB2,STAT1	3.5	0.0405		APOE,LYZ,ALB,APOA1,APOB,RAB7A
Communication between Innate and Adaptive Immune Cells	4.76	0.0833		TLR2,HLA-DRB1,HLA-A,HLA-B,HLA-DRA,FCER1G,IL1B,HLA-DRB5	3.49	0.0521		HLA-DRB1,HLA-A,HLA-B,IL1B,HLA-DRB5
Th2 Pathway	2.04	0.0395		HLA-DRB1,ICAM1,HLA-A,HLA-B,HLA-DRA,HLA-DRB5	3.44	0.0395		ITGB2,HLA-DRB1,ICAM1,HLA-A,HLA-B,HLA-DRB5
IL-4 Signaling	2.96	0.0606		SHC1,HLA-DRB1,HLA-A,HLA-B,HLA-DRA,HLA-DRB5	3.43	0.0505		HLA-DRB1,HLA-A,HLA-B,HMGA1,HLA-DRB5
Tumoricidal Function of Hepatic Natural Killer Cells	1.5	0.0833		ICAM1,ITGAL	3.38	0.125		ICAM1,CYCS,ITGAL
Cdc42 Signaling	3.79	0.0539	0	MYL9,HLA-DRB1,WAS,HLA-A,HLA-B,HLA-DRA,FCER1G,HLA-DRB5,IQGAP3	3.22	0.0359		HLA-DRB1,HLA-A,HLA-B,ITGA5,HLA-DRB5,IQGAP3
Calcium-induced T Lymphocyte Apoptosis	3.91	0.0909	2.236	HLA-DRB1,HLA-A,HLA-B,HLA-DRA,FCER1G,HLA-DRB5	3.13	0.0606	0	HLA-DRB1,HLA-A,HLA-B,HLA-DRB5
Cytotoxic T Lymphocyte-mediated Apoptosis of Target Cells	2.21	0.0938		HLA-A,HLA-B,FCER1G	3	0.0938		HLA-A,HLA-B,CYCS
T Helper Cell Differentiation	4.63	0.0959		HLA-DRB1,HLA-A,HLA-B,HLA-DRA,FCER1G,STAT1,HLA-DRB5	2.97	0.0548		HLA-DRB1,HLA-A,HLA-B,HLA-DRB5
Th1 and Th2 Activation Pathway	2.18	0.0374		ICAM1,HLA-DRB1,HLA-A,HLA-B,HLA-DRA,STAT1,HLA-DRB5	2.97	0.0321		ITGB2,HLA-DRB1,ICAM1,HLA-A,HLA-B,HLA-DRB5
Coagulation System	3.14	0.114	-2	KNG1,SERPINC1,A2M,SERPINF2	2.89	0.0857		KNG1,SERPINC1,SERPINF2
Production of Nitric Oxide and Reactive Oxygen Species in Macrophages	3.81	0.049	1.897	TLR2,PPP2CB,APOE,LYZ,NCF1,ALB,APOB,APOA1,NFKB2,STAT1	2.78	0.0294	0	APOE,LYZ,NCF1,ALB,APOA1,APOB
Allograft Rejection Signaling	3.28	0.0698		HLA-DRB1,HLA-A,HLA-B,HLA-DRA,FCER1G,HLA-DRB5	2.71	0.0465		HLA-DRB1,HLA-A,HLA-B,HLA-DRB5
OX40 Signaling Pathway	4.04	0.0778		HLA-DRB1,HLA-A,HLA-B,HLA-DRA,FCER1G,NFKB2,HLA-DRB5	2.63	0.0444		HLA-DRB1,HLA-A,HLA-B,HLA-DRB5
Reelin Signaling in Neurons	1.61	0.0426		APOE,YES1,HCK,ITGAL	2.56	0.0426		APOE,ITGB2,ITGA5,ITGAL
Inflammasome pathway	4.11	0.2	2	CASP1,CTSB,IL1B,NFKB2	2.18	0.1		CTSB,IL1B
Role of NFAT in Regulation of the Immune Response	2.07	0.0357	2.646	HLA-DRB1,HLA-A,HLA-B,HLA-DRA,FCER1G,NFKB2,HLA-DRB5	2.13	0.0255	-0.447	BTK,HLA-DRB1,HLA-A,HLA-B,HLA-DRB5
iCOS-iCOSL Signaling in T Helper Cells	3.94	0.064	2.646	SHC1,HLA-DRB1,HLA-A,HLA-B,HLA-DRA,FCER1G,NFKB2,HLA-DRB5	2.12	0.032	0	HLA-DRB1,HLA-A,HLA-B,HLA-DRB5

CD28 Signaling in T Helper Cells	3.73	0.0597		HLA-DRB1,HLA-A,WAS,HLA-B,HLA-DRA,FCER1G,NFKB2,HLA-DRB5	2.02	0.0299		HLA-DRB1,HLA-A,HLA-B,HLA-DRB5
Osteoarthritis Pathway	1.89	0.033	2.646	TLR2,PPARD,CASP1,IL1B,NAMPT,NFKB2,MMP9	2	0.0236	1.342	MATN3,PPARD,IL1B,ITGA5,MMP9
TREM1 Signaling	3.6	0.08	2.449	TLR2,ICAM1,TYROBP,CASP1,IL1B,NFKB2	1.95	0.04		ICAM1,IL1B,ITGA5
Phagosome Formation	1.61	0.0357		TLR2,MARCKS,C5AR1,VTN,FCER1G	1.95	0.0286		ITGB2,MARCKS,C5AR1,ITGA5
Systemic Lupus Erythematosus Signaling	1.64	0.0293		KNG1,LSM6,SNRPB,HLA-A,HLA-B,FCER1G,IL1B	1.79	0.0209		KNG1,HLA-A,HLA-B,IL1B,SNRNP40
Fcγ Receptor-mediated Phagocytosis in Macrophages and Monocytes	3.07	0.0638	1.633	HMOX1,YES1,NCF1,WAS,ACTB,HCK	1.69	0.0319		HMOX1,NCF1,ACTB
PKCθ Signaling in T Lymphocytes	2.4	0.0412	2.646	HLA-DRB1,HLA-A,HLA-B,HLA-DRA,FCER1G,NFKB2,HLA-DRB5	1.68	0.0235	0	HLA-DRB1,HLA-A,HLA-B,HLA-DRB5
Hepatic Fibrosis / Hepatic Stellate Cell Activation	2.79	0.043		MYL9,COL6A1,ICAM1,IL1B,NFKB2,STAT1,A2M, MMP9	1.55	0.0215		ICAM1,COL6A1,IL1B,MMP9
T Cell Exhaustion Signaling Pathway	3.37	0.0474	1	PPP2CB,HLA-DRB1,HLA-A,HLA-B,HLA-DRA,FCER1G,EOMES,STAT1,HLA-DRB5	1.52	0.0211		HLA-DRB1,HLA-A,HLA-B,HLA-DRB5
Cell Cycle Control of Chromosomal Replication	2.38	0.0714		ORC2,RPA3,TOP2A,MCM4	1.33	0.0357		ORC2,RPA3
Agrin Interactions at Neuromuscular Junction					3.92	0.0641	1	ITGB2,PKLR,ACTB,ITGA5,ITGAL
Granulocyte Adhesion and Diapedesis					3.91	0.0391		ITGB2,ICAM1,C5AR1,IL1B,ITGA5,ITGAL,MMP9
Neuroprotective Role of THOP1 in Alzheimer's Disease					3.05	0.0417		KNG1,HLA-A,HLA-B,PRSS1,MMP9
LPS/IL-1 Mediated Inhibition of RXR Function					2.57	0.0268		APOE,HS2ST1,IL1B,FABP4,PAPSS2,IL4I1
Mitochondrial Dysfunction					2.38	0.0292		SOD2,ATP5F1D,COX7C,CYCS,ATP5ME
Oxidative Phosphorylation					2.33	0.0367	2	ATP5F1D,COX7C,CYCS,ATP5ME
Paxillin Signaling					2.14	0.0323		ITGB2,ACTB,ITGA5,ITGAL
Remodeling of Epithelial Adherens Junctions					2.05	0.0435		ACTB,TUBA4A,RAB7A
NF-κB Activation by Viruses					1.64	0.0306		ITGB2,ITGA5,ITGAL
Sirtuin Signaling Pathway					1.46	0.0171	-1	SOD2,PPIF,ATP5F1D,GLS,TUBA4A
NRF2-mediated Oxidative Stress Response					1.41	0.0194		HMOX1,SOD2,ACTB,SQSTM1
IL-8 Signaling					1.36	0.0186	2	ITGB2,HMOX1,ICAM1,MMP9
Rac Signaling					1.36	0.0236		CD44,ITGA5,IQGAP3
Interferon Signaling	9.59	0.25	3	IFIT1,IFIT3,IFITM3,OAS1,MX1,IFI35,STAT1,TAP1, ISG15				
Glucocorticoid Receptor Signaling	4.74	0.0429		KRT14,ICAM1,ACTB,KRT9,KRT5,PTGES3,SHC1,KRT13,KRT6A,IL1B,KRT2,STAT1,KRT1,A2M, KRT10				

Role of Pattern Recognition Receptors in Recognition of Bacteria and Viruses	3.75	0.0533	2.646	TLR2,OAS1,C3,C5AR1,DDX58,CASP1,IL1B,NFKB2,OAS3	
Synaptogenesis Signaling Pathway	2.26	0.0306	2.333	NAP1L4,APOE,MARCKS,SHC1,RAB5A,YES1, COMP,WAS,HCK,NAPA	
Tec Kinase Signaling	2.26	0.0389	1.89	YES1,WAS,ACTB,HCK,FCER1G,NFKB2,STAT1	
Fatty Acid β-oxidation I	2.21	0.0938		ACSL4,ACAA2,ACSL1	
Activation of IRF by Cytosolic Pattern Recognition Receptors	2.19	0.0635	1	DDX58,NFKB2,STAT1,ISG15	
CTLA4 Signaling in Cytotoxic T Lymphocytes	2.17	0.0495		PPP2CB,HLA-A,HLA-B,FCER1G,AP1G1	
Tight Junction Signaling	1.84	0.0357		MYL9,PPP2CB,ACTB,CPSF1,NFKB2,NAPA	
Protein Ubiquitination Pathway	1.82	0.0295		UBE2A,CDC20,HLA-A,HLA-B,TAP2,TAP1,DNAJB14,HSPA4L	
EIF2 Signaling	1.7	0.0302	1	EIF3G,SHC1,WARS,ACTB,RPL10,EIF4G3,RPL36	
ILK Signaling	1.47	0.0293	-0.447	MYL9,FLNB,PPP2CB,ACTB,NFKB2,MMP9	
PPAR Signaling	1.47	0.0385	-2	SHC1,PPARD,IL1B,NFKB2	
Autophagy	1.46	0.0492		CTSS,CTSB,SQSTM1	
IL-10 Signaling	1.32	0.0435		HMOX1,IL1B,NFKB2	
